# Antibody–Drug Conjugates (ADCs): A Review of Structural Design, Technological Evolution, and Future Perspectives

**DOI:** 10.3390/molecules31071180

**Published:** 2026-04-02

**Authors:** Guiying Wu, Zhenhai Yuan, Ming Chen, Xuan Tang, Fang Wang, Daizhou Zhang

**Affiliations:** Institute of Traditional Chinese Medicine, Shandong Academy of Pharmaceutical Sciences, Jinan 250101, China; wuguiying@sdaps.cn (G.W.); yuanzhenhai@sdaps.cn (Z.Y.); chenming@sdaps.cn (M.C.); tangxuan@sdaps.cn (X.T.)

**Keywords:** antibody–drug conjugates, monoclonal antibody, linker, cytotoxic payload

## Abstract

Antibody–drug conjugates (ADCs) have become an important class of targeted anticancer therapeutics by integrating the tumor selectivity of monoclonal antibodies with the potent cytotoxicity of small-molecule payloads through rational linker design. This review summarizes the structural fundamentals of ADCs, including antibodies, linkers, and payloads, and describes their coordinated mechanism of action. We trace the evolutionary trajectory of ADCs across three generations, highlighting key breakthroughs, limitations, and representative agents for each era. Furthermore, we elaborate on cleavage mechanisms of linkers (cleavable and non-cleavable). We also categorize and discuss cytotoxic payloads, covering traditional microtubule-disrupting agents, DNA-damaging agents, and novel mechanism-based payloads, along with their modification strategies and preclinical/clinical performance. Finally, we discuss representative and clinically influential ADC designs, with emphasis on the relationships among antibody, linker, and payload.

## 1. Introduction

Malignant tumors remain a major global health burden, and the incidence and mortality of many cancers continue to rise despite advances in surgery, radiotherapy, chemotherapy, targeted therapy, and immunotherapy. According to GLOBOCAN statistics, cancer caused nearly 10 million deaths worldwide in 2018 and continues to pose an alarming threat to public health [1]. Traditional chemotherapeutic agents, although often highly potent, suffer from intrinsic limitations such as poor tumor selectivity, systemic toxicity, and dose-limiting adverse effects. These drawbacks significantly restrict their clinical utility and frequently lead to suboptimal therapeutic outcomes, especially in metastatic or refractory cancers. With the rapid development of molecular oncology, precision medicine, and antibody engineering, researchers have increasingly sought to design therapeutic strategies that combine high selectivity with potent cytotoxicity. Among these emerging modalities, antibody–drug conjugates (ADCs) have become one of the most promising classes of targeted anticancer agents. Conceptually rooted in Paul Ehrlich’s early “magic bullet” theory proposed more than a century ago [2], ADCs deliver cytotoxic payloads directly to tumor cells via monoclonal antibodies that recognize tumor-associated antigens, thereby enable precise tumor killing while sparing healthy tissues.

Since the approval of the first ADC, gemtuzumab ozogamicin (Mylotarg), in 2000 for the treatment of acute myeloid leukemia, ADC technology has experienced remarkable growth and transformation [3,4]. Early-generation ADCs were hampered by the use of murine antibodies, unstable linkers, and insufficiently potent cytotoxic drugs, resulting in unpredictable pharmacokinetics, off-target toxicity, and limited therapeutic activity [5,6]. However, subsequent advances in antibody humanization, linker chemistry, and ultra-potent payload development have significantly improved the stability, specificity, and efficacy of ADCs. Over the past decade, ADCs have rapidly evolved into a major therapeutic class in oncology, with 16 ADCs approved globally as of 2024 and more than 425 candidates currently in clinical development [4]. These agents have demonstrated impressive clinical benefits in hematologic malignancies and a growing number of solid tumors, including breast cancer, ovarian cancer, urothelial carcinoma, non-small cell lung cancer, and cervical cancer [7,8,9,10]. The success of representative ADCs such as enfortumab vedotin (Padcev), trastuzumab deruxtecan (Enhertu), and sacituzumab govitecan (Trodelvy) has reshaped treatment paradigms and expanded the therapeutic landscape for patients with limited treatment options [11,12].

The rapid expansion of the ADC field is driven by its inherently multidisciplinary nature. The design of ADCs requires an intricate integration of antibody biology, medicinal chemistry, bioconjugation strategies, cellular trafficking mechanisms, pharmacokinetic modeling, and manufacturability considerations. The antibody component determines antigen specificity, internalization behavior, and Fc-mediated immune effector functions [13]. The linker governs systemic stability and intracellular payload release, balancing the conflicting demands of circulation stability and efficient drug liberation [14]. The payload, typically 100–1000 times more potent than classical chemotherapeutics [15], must be structurally compatible with conjugation and able to exert sustained intracellular cytotoxicity. Meanwhile, both the bioconjugation method and the resulting drug-to-antibody ratio (DAR) profoundly influence ADC pharmacokinetics, aggregation propensity, and therapeutic index [16,17,18,19]. Even subtle structural modifications—such as a single linker substitution or a shift in conjugation site—may drastically alter ADC behavior in vivo. Consequently, ADC development must treat the molecule as an integrated system rather than a simple combination of three modular components.

Despite the significant progress achieved, the clinical translation of ADCs continues to face challenges. Tumor antigen heterogeneity can limit the therapeutic reach of ADCs and contribute to primary or acquired resistance [20]. Off-target toxicity remains a major concern, particularly for ADCs with unstable linkers or highly membrane-permeable payloads, which may cause systemic adverse effects such as hepatotoxicity, myelosuppression, or neuropathy. The complexity of intracellular trafficking, involving diverse endocytic routes and variable lysosomal activity, influences payload release efficiency and therapeutic potency. Furthermore, many tumor cells upregulate efflux transporters such as P-glycoprotein, reducing intracellular payload accumulation. Manufacturing high-quality ADCs with precise DAR control and adequate stability also remains challenging, especially for site-specific conjugation technologies [21]. These limitations have driven efforts to develop next-generation ADCs featuring improved antigen selection strategies, bispecific or biparatopic antibody formats, cleavable linkers responsive to tumor-specific stimuli such as proteases, acidic pH, or ROS [22,23,24,25], and entirely new classes of cytotoxic drugs, including RNA splicing inhibitors [26], Bcl-xL inhibitors [27], and NAMPT inhibitors [28].

As ADC technologies continue to mature, their clinical impact is rapidly expanding. Several new-generation ADCs have demonstrated not only potent cytotoxic activity but also the ability to modulate antitumor immunity, including induction of immunogenic cell death and synergy with immune checkpoint inhibitors [29]. Notably, trastuzumab deruxtecan has shown significant benefit in HER2-low tumors, challenging long-standing biomarker definitions and broadening the scope of targeted therapy [30]. In addition, extracellular-cleavable linkers and high-bystander-effect payloads have shown promise in treating tumors with low internalization capacity or marked intratumoral heterogeneity. These advancements suggest that ADCs are transitioning toward highly engineered “precision drug delivery systems” capable of exploiting tumor biology at multiple levels.

Given the complexity and rapid evolution of the ADC field, a focused review is warranted to synthesize the key design principles that have shaped clinically relevant progress. Rather than attempting to exhaustively catalogue every reported ADC, the present review highlights representative and influential examples to discuss the structural fundamentals of ADCs, their mechanism of action, technological evolution, clinically validated and emerging targets, linker chemistry, payload classes, and ongoing challenges. By emphasizing the relationships among antibody format, linker design, and payload properties, this article aims to support the rational development of next-generation ADCs with improved efficacy, safety, and translational potential.

## 2. Mechanism of Action of ADCs

**Figure 1:**
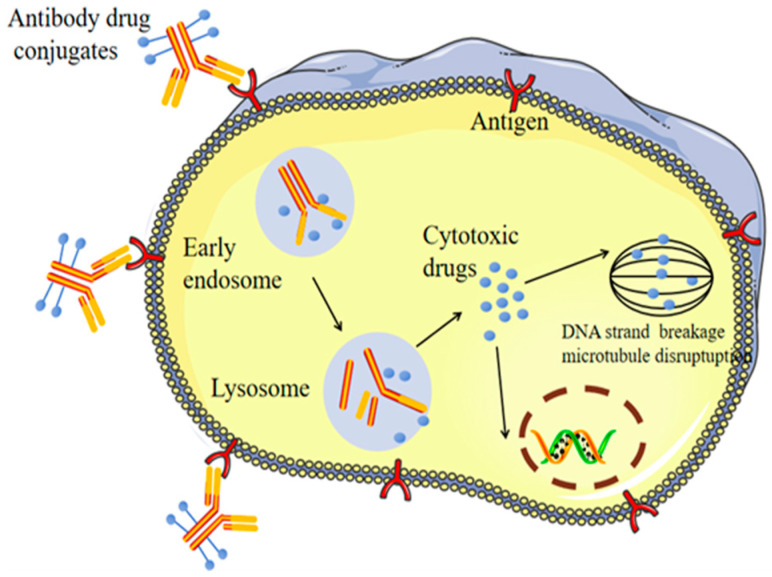
The structure and characteristics of ADC.

After intravenous administration, ADCs enter the systemic circulation and distribute throughout the body. Owing to the high specificity of the monoclonal antibody (mAb) component, ADCs selectively recognize and bind to cognate antigens overexpressed on the surface of tumor cells, forming a stable antigen–ADC complex. This complex is then internalized via receptor-mediated endocytosis. Following internalization, several intracellular fates are possible: (i) FcRn-mediated recycling—A fraction of internalized ADC–antigen complexes interacts with neonatal the Fc receptor (FcRn) in the endosomal compartment [31]. These complexes can be recycled back to the cell surface and released into the circulation, representing a physiological “buffering” process that may partially mitigate off-target uptake by normal cells. (ii) Lysosomal trafficking and payload release—The remaining ADC–antigen complexes are trafficked to lysosomes. Within this protease-rich and often acidic environment, the antibody backbone is degraded and/or the linker is cleaved. For non-cleavable linkers, lysosomal proteases digest the antibody to liberate an active drug–amino acid adduct. For cleavable linkers, chemical or enzymatic triggers (e.g., acidic pH, redox potential, lysosomal proteases, and glycosidases) promote linker scission, resulting in the release of the active cytotoxic payload. The released payload then exerts its pharmacological effect by interfering with essential cellular processes, such as microtubule dynamics, DNA replication and repair, topoisomerase function, or transcription [32]. This ultimately leads to cell-cycle arrest and apoptosis (Figure 1). Depending on the membrane permeability of the released species, certain ADCs can also induce a “bystander effect,” whereby the liberated payload diffuses into neighboring antigen-low or antigen-negative tumor cells.

## 3. Evolution of ADC Technology

First-generation ADCs predominantly employed murine monoclonal antibodies, with Pfizer’s Mylotarg (gemtuzumab ozogamicin) serving as a representative example for the treatment of CD33-positive acute myeloid leukemia (AML) [13]. However, due to significant safety concerns, including severe toxicity, Mylotarg was ultimately withdrawn from the market in 2010 [33,34]. The agent was later re-approved in 2017, including in combination with chemotherapy, for the treatment of AML [34]. Another illustrative case is BR96-doxorubicin, which was constructed by conjugating a conventional chemotherapeutic agent, doxorubicin, to a murine antibody via a non-cleavable linker. Overall, these early ADCs did not demonstrate superior efficacy relative to their corresponding free cytotoxic agents. Moreover, their linker chemistry was found to undergo slow hydrolysis in systemic circulation, resulting in uncontrolled payload release and unexpected off-target toxicity. The limitations of first-generation ADCs catalyzed the development of second-generation ADCs with improved antibody engineering, linker stability, and payload design [29,35].

At present, the majority of marketed ADCs are considered second-generation products. Representative examples include brentuximab vedotin for relapsed Hodgkin lymphoma and systemic anaplastic large cell lymphoma, ado-trastuzumab emtansine (T-DM1) for HER2-positive metastatic breast cancer [14,15,36], and gemtuzumab ozogamicin for acute myeloid leukemia. In addition, inotuzumab ozogamicin and the more recently approved polatuzumab vedotin-piiq for B-cell malignancies were designed as potent anticancer agents that enable tumor-selective delivery of highly cytotoxic payloads while sparing healthy tissues [17,37]. By coupling these ultrapotent cytotoxins to tumor-specific antibodies, their lethal activity can be concentrated within antigen-expressing tumor cells. Moreover, antibody conjugation can improve the pharmacokinetic behavior of small-molecule payloads, effectively conferring an immunoglobulin-like prolonged half-life relative to the unconjugated drugs. Despite these advances, second-generation ADCs still face important limitations, including off-target toxicity, premature clearance (in certain settings), and competition with the unconjugated antibody for antigen binding and tumor penetration.

Lessons learned from earlier ADC generations have accelerated the development of third-generation ADCs, whose major innovations center on conjugation chemistry and control of drug loading. In particular, site-specific conjugation enables the production of ADCs with defined and homogeneous drug-to-antibody ratios (DARs), most commonly DAR 2 or DAR 4, thereby improving product consistency, reducing off-target toxicity, and enhancing pharmacokinetic performance. In parallel, continued exploration of novel ADC targets and next-generation payload classes is further expanding therapeutic opportunities and improving antitumor efficacy [38,39].

Beyond improvements in payload potency and linker stability, advances in conjugation chemistry have also become a defining feature of modern ADC development. As ADC design has matured, it has become increasingly evident that linker chemistry cannot be considered in isolation from conjugation chemistry. In early ADCs, payloads were commonly installed through stochastic coupling to solvent-accessible lysine residues or to cysteines exposed after partial reduction of interchain disulfides [39,40]. These approaches are experimentally straightforward and, in some settings, remain serviceable. Their limitation, however, is inherent: they generate mixtures that differ not only in drug-to-antibody ratio (DAR), but also in the positional distribution of the payload on the antibody surface [39]. This heterogeneity is not merely a manufacturing inconvenience. A series of influential studies showed that the conjugation site can materially affect hydrophobicity, aggregation propensity, deconjugation kinetics, plasma exposure, and ultimately the therapeutic index [41,42]. In other words, the site of attachment is itself a design variable, rather than a passive consequence of synthetic accessibility [42].

This recognition drove the development of site-specific conjugation strategies [32,39]. Among the earliest approaches to demonstrate clear translational value were engineered-cysteine platforms, exemplified by THIOMAB-type constructs, which introduced predefined thiol-bearing sites and thereby enabled the preparation of ADCs with tighter DAR distributions and improved in vivo performance [41,42,43]. Subsequent work made an equally important point: not all engineered sites behave similarly. Even when the same linker–payload is used, local microenvironment, solvent accessibility, and susceptibility of the maleimide linkage to exchange or hydrolysis can lead to marked differences in stability and pharmacokinetics [42,43]. These studies shifted the field away from a purely stoichiometric view of conjugation and toward a more structural one, in which DAR control and site selection are optimized together.

A broader set of orthogonal technologies has since expanded the scope of site-selective ADC assembly [39,43,44,45]. Genetically encoded noncanonical amino acids offer uniquely reactive handles and permit precise control over both site and stoichiometry [44]. Enzyme-mediated methods, including microbial transglutaminase- and sortase-based conjugation, provide efficient routes to homogeneous constructs under comparatively mild conditions [44,45]. Collectively, these approaches are valuable not because they produce analytically cleaner materials per se, but because they allow conjugation site, linker stability, and payload properties to be tuned in a coordinated manner [39,42]. In current ADC development, linker attachment chemistry is therefore best regarded as a central component of molecular design, closely linked to developability, pharmacology, and safety, rather than as a downstream technical refinement [41,42].

## 4. Antibody Targets

The antibody component of an ADC primarily mediates target recognition and can trigger target antigen-dependent internalization from the tumor cell surface. Among the available antibody formats, humanized immunoglobulin G (IgG) is the most widely used in ADCs owing to its comparatively low immunogenicity and favorable developability. IgG comprises four subclasses (IgG1, IgG2, IgG3, and IgG4) [46]. Notably, among currently approved ADCs, only a small minority employ IgG4 as the antibody scaffold, whereas the vast majority utilize IgG1. This preference is largely attributable to the superior in vivo stability and longer serum half-life of IgG1, together with its higher affinity for Fc gamma receptors (FcγRs), which facilitates more efficient engagement with innate immune effector cells such as natural killer (NK) cells and macrophages [46,47,48].

However, preservation of full Fc-mediated effector function is not always desirable in ADC design. For many ADCs, the principal therapeutic objective is selective intracellular delivery of the payload, rather than recruitment of immune effector function in the manner expected for a naked antibody [49,50]. Under these circumstances, Fc engagement with Fcγ receptors or C1q can be a mixed blessing. On the one hand, such interactions may support immune cell engagement; on the other hand, they may alter biodistribution, promote uptake by FcγR-positive myeloid populations, or increase non-productive clearance [50]. These considerations are particularly relevant for ADCs carrying highly potent or membrane-permeable payloads, where even limited target-independent handling may have toxicologic consequences [50].

This is the rationale for growing interest in Fc-engineered or Fc-silent antibodies. A range of Fc-silencing solutions has been reported, including LALA-type, LALE-type, and other variants designed to attenuate Fcγ receptor and/or complement binding while preserving antigen recognition [50,51]. More recent comparative work has also emphasized that Fc silencing should not be judged solely by loss of effector function; developability remains equally important [51]. Some variants show more favorable stability, manufacturability, and overall biophysical behavior than others, indicating that Fc silencing is not a single uniform design choice but a family of related engineering solutions that must be selected with care [51]. From an ADC perspective, these formats are particularly attractive when the antibody is intended primarily as a delivery vehicle rather than as an effector-function-bearing therapeutic agent in its own right [49,51].

At the same time, Fc silencing should not be presented as a universally superior design rule [49,50,52]. Preclinical studies have suggested that, in some tumor-associated macrophage-rich settings, Fc–FcγR interactions may contribute to intratumoral ADC processing and can augment antitumor activity [52]. Conversely, other work has shown that FcγR-dependent internalization of ADC aggregates can enhance off-target cytotoxicity in FcγR-expressing cells, and that Fc silencing can mitigate this liability [50]. The literature therefore supports a fit-for-purpose view of Fc engineering in ADC development [48,49,50,51,52]. Full IgG1 effector function, attenuated Fc engagement, and deeply silenced Fc variants should each be viewed as context-dependent options, to be selected according to target biology, tumor microenvironment, payload class, and safety priorities rather than by default convention.

An ideal ADC antibody should bind tumor-selective antigens with high specificity, ideally antigens that are uniquely expressed on tumor cells and absent (or expressed at negligible levels) on normal tissues, to maximize tumor targeting while minimizing on-target/off-tumor toxicity.

As of October 2025, the 16 marketed ADCs span 11 distinct targets (Table 1), including CD33, CD30, HER2, CD22, CD79b, Nectin-4, BCMA, EGFR, CD19, tissue factor (TF), and folate receptor-α (FRα) [8,53]. Among the approximately 425 ADC candidates currently in development, the most intensively pursued targets include HER2, EGFR, CLDN18.2, Trop-2, c-MET, CD19, PSMA, MUC1, BCMA, and PD-L1. Many of these are well-established targets with substantial clinical validation, whereas others remain under active clinical investigation. In addition, a subset of programs is advancing bispecific or multispecific formats, such as HER2 bispecific antibodies, PD-L1/PD-L2 bispecific antibodies, and CLDN9/CLDN6 bispecific antibodies.

Hematologic malignancies are typically characterized by aberrant overexpression of lineage-associated surface markers, including CD19, CD20, CD22, CD33, BCMA, and CD79 [53,54]. CD19, a canonical marker of the B-cell lineage, is expressed across much of B-cell ontogeny, from early (pre-) B-cell stages through differentiation, and is also detectable on follicular dendritic cells [55,56]. As a key co-receptor within the B-cell receptor (BCR) signaling complex, CD19 has been well established as a central therapeutic target in B-cell non-Hodgkin lymphoma (B-NHL) and B-cell leukemias. CD20 is predominantly localized on the surface of naïve and mature B lymphocytes and is lost during terminal differentiation toward plasma cells; its frequent high expression in B-cell lymphomas makes it an important target for disease intervention.

CD22 primarily functions as a negative regulator of BCR signal transduction and contributes to B-cell trafficking and the maintenance of peripheral B-cell immune tolerance. It is broadly expressed on normal B cells and is also present on differentiated B-cell populations in B-acute lymphoblastic leukemia (B-ALL), as well as on the majority of B-ALL progenitor cells [57,58]. CD33 shows a myeloid progenitor-restricted expression pattern, with expression typically decreasing during maturation; however, its persistent expression on leukemic blasts in acute myeloid leukemia (AML) and in subsets of acute lymphoblastic leukemia (ALL)-associated cells supports its utility as a key marker and target for hematologic tumor-directed therapy. BCMA (B-cell maturation antigen), a member of the tumor necrosis factor receptor (TNFR) superfamily, regulates the survival of plasmablasts and plasma cells in the bone marrow through ligand-dependent signaling [23,59]. This axis also plays a pivotal role in the malignant proliferation of multiple myeloma, making BCMA a particularly compelling therapeutic target [23,60]. CD79, a heterodimer composed of CD79a and CD79b, is an essential component of the BCR signaling complex; notably, CD79b is selectively expressed on B cells and is enriched on the surface of certain B-cell lymphoma cells (including Burkitt lymphoma and other B-NHL subtypes), supporting its consideration as a high-value target for precision therapy [61].

In solid tumors, therapeutic antibodies most commonly target receptor molecules that are aberrantly overexpressed on the surface of malignant cells, such as HER2, TROP2, Nectin-4, FRα, and tissue factor (TF) [62,63,64,65]. HER2 (human epidermal growth factor receptor 2) is frequently upregulated at the plasma membrane across multiple malignancies, including biliary tract cancer, colorectal cancer, non-small cell lung cancer (NSCLC), bladder cancer, and breast cancer [65,66]. In breast cancer, approximately 10–20% of cases exhibit HER2 overexpression, which can directly enhance tumor cell proliferation and survival. Importantly, HER2 is not exclusively tumor-restricted; it is also physiologically expressed on the membrane of normal epithelial tissues and cardiomyocytes, a feature that may contribute to cardiotoxicity associated with HER2-targeted therapies [67].

TROP2 is a cell surface glycoprotein whose dysregulated expression in solid tumors has made it an attractive target for precision therapy. While TROP2 shows physiological expression in epithelial compartments of several healthy tissues, including skin, cervical epithelium, tonsil, and thymus, it is also expressed across diverse epithelial-derived cancers such as breast, pancreatic, urothelial, and ovarian cancers. Upon binding growth factors that promote survival and proliferation, TROP2 can initiate downstream signaling cascades that regulate key oncogenic behaviors [68].

Nectin-4, a type I transmembrane protein and a member of the nectin immunoglobulin-like adhesion molecule family, primarily mediates calcium-independent cell–cell adhesion and contributes to cytoskeletal remodeling. Nectin-4 expression is generally low in normal tissues, whereas its marked upregulation is a characteristic molecular feature of multiple epithelial malignancies [69].

Folate receptor-α (FRα) is a membrane-associated receptor with high affinity for folate and several folate derivatives, and it is frequently overexpressed on the surface of ovarian, lung, and breast cancer cells. Beyond mediating folate internalization, FRα-associated trafficking has been implicated in the transcriptional regulation of growth-promoting pathways; notably, folate transport is essential for DNA biosynthesis, supporting the rationale for FRα-directed targeting strategies [70,71].

Tissue factor (TF), also known as coagulation factor III or CD142, is a procoagulant transmembrane glycoprotein that forms a complex with factor VIIa and can trigger intracellular signaling. Under physiological conditions, TF is expressed on the surface of monocytes, platelets, and epithelial cells. In contrast, TF is aberrantly upregulated in a range of malignancies, including glioblastoma, breast cancer, colorectal cancer, pancreatic cancer, lung cancer, and cervical cancer, where it has emerged as a prominent molecular feature with therapeutic relevance [72].

In antibody-based targeted therapy, programmed cell death protein 1 (PD-1) and its ligand PD-L1 are among the most well-established immune checkpoint biomarkers and therapeutic targets [73]. Unlike most conventional targeted agents that primarily act on tumor-intrinsic signaling pathways, inhibitors of the PD-1/PD-L1 axis exhibit a dual targeting profile: they may act directly on tumor cells that express PD-L1 while simultaneously modulating immune cell function by blocking inhibitory signaling, thereby relieving immune suppression and restoring cytotoxic effector activity.

At present, clinical trials of ADCs continue to expand into novel target spaces, with ongoing programs directed against emerging tumor-associated antigens such as claudin-6 (CLDN6), delta-like ligand 3 (DLL3), mesothelin (MSLN), the zinc transporter LIV-1 (SLC39A6), and B7 homolog 4 (B7-H4), among others.

## 5. Linkers

An ideal linker should exhibit robust circulatory stability to prevent premature payload release in the bloodstream, while still enabling controlled payload liberation once the conjugate reaches its intended site of action. The release trigger mechanism embedded within the linker largely determines whether payload release is “stimulus-responsive.” For cleavable linkers, specific chemical triggers are designed to initiate payload release upon exposure to defined biological stimuli, such as lysosomal enzymes, acidic pH, or redox conditions [74,75]. In contrast, non-cleavable linkers lack such triggers and function primarily as stable covalent tethers that anchor the payload to the antibody. Accordingly, linkers are commonly categorized into two major classes: non-cleavable and cleavable linkers.

### 5.1. Non-Cleavable Linkers

#### Thioether Linkers

**Figure 2:**
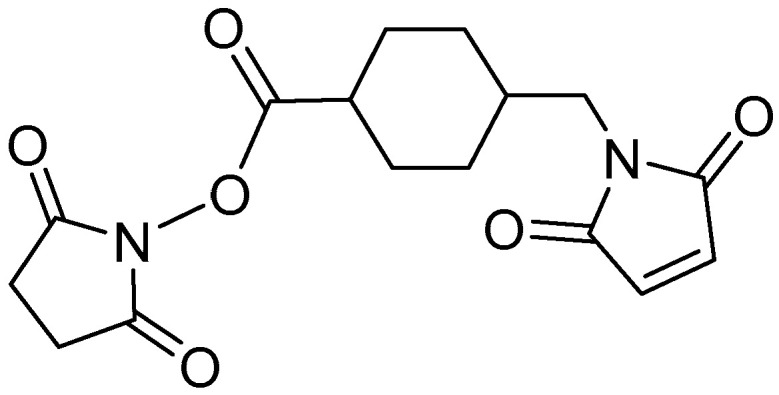
Structure of non-cleavable thioether linker SMCC used in ADC construction.

ADCs incorporating non-cleavable linkers generally rely on cellular internalization for activity, as payload release occurs only after the antibody component undergoes proteolytic degradation within lysosomes [75,76]. Over the course of ADC development, multiple non-cleavable linker chemistries have been established, among which N-succinimidyl 4-(N-maleimidomethyl)cyclohexane-1-carboxylate (SMCC) is a prototypical example (Figure 2). The marketed ADC Kadcyla (ado-trastuzumab emtansine; T-DM1) employs an SMCC-derived thioether linkage, which confers excellent stability in systemic circulation. Compared with earlier cleavable linker designs, this architecture is associated with reduced premature payload release and consequently lower off-target toxicity. In addition, the SMCC-based linker is structurally straightforward, supports controllable manufacturing, and is particularly well suited for conjugation with maytansinoid payloads (e.g., DM1/DM4).

Following intracellular processing and catabolism, ADCs bearing SMCC-type non-cleavable linkers predominantly yield Lys–SMCC–DM1 as a characteristic tumor-associated catabolite [77]. Notably, because this released catabolite exhibits limited membrane permeability, ADCs built on non-cleavable linker designs typically show minimal bystander killing, as the active species is less able to diffuse into neighboring antigen-negative cells.

### 5.2. Cleavable Linkers

Cleavable linkers can be further classified according to their payload-release mechanisms into two broad categories: enzyme-dependent and chemical triggered linkers. In both cases, release is governed by the physicochemical characteristics of the cleavage site and the relevant biological milieu, most commonly the lysosomal compartment and/or the tumor microenvironment [77,78].

#### 5.2.1. Chemically Triggered Linkers

##### Disulfide Linkers

Disulfide-containing linkers can undergo cleavage via thiol-mediated nucleophilic attack, resulting in release of the active payload. Although reduced human serum albumin (HSA) constitutes the most abundant thiol species in plasma, its reactivity toward macromolecular disulfides is relatively limited. In contrast, the cytosol contains high concentrations of glutathione (GSH), a thiol-bearing tripeptide, with substantially greater nucleophilic reactivity toward disulfide bonds [76,77]. Importantly, GSH levels are typically in the micromolar range in blood but reach the millimolar range intracellularly; this pronounced gradient, together with oxidative stress frequently associated with cancer cells, favors preferential intracellular payload release within tumor cells.

**Figure 3:**
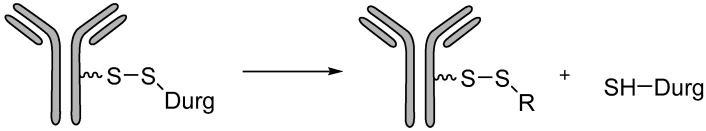
Structure of the SAR3419 linker–payload module (SPDB–DM4), highlighting the sterically hindered disulfide linker.

Disulfide linkers are most commonly paired with maytansinoid payloads, and their reactivity can be tuned through steric shielding. For example, α-methyl substitution can markedly alter the reduction rate and increase resistance to thiol–disulfide exchange. A representative case is SAR3419, in which a sterically hindered disulfide linker incorporating gem-dimethyl substitution was optimized to generate the SPDB-DM4 construct, ultimately achieving improved antitumor activity (Figure 3). In Figure 3, the antibody-reactive terminus of the linker and the DM4-bearing end are indicated separately to clarify how the SPDB-DM4 module is assembled onto the antibody scaffold.

##### Hydrazone Linkers

**Scheme 1:**
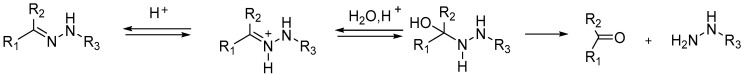
Acid-catalyzed hydrolysis of a hydrazone linker under endosomal/lysosomal conditions.

Hydrazone linkers exhibit pH-dependent stability: they remain relatively stable under neutral conditions but undergo acid-catalyzed hydrolysis in acidic compartments, such as endosomes (pH < 6) and lysosomes (pH < 5), to generate the corresponding ketone and hydrazide products (Scheme 1).

Hydrazone linkers have also been widely used with calicheamicin-family payloads, in which payload liberation proceeds through a two-step activation process. First, the acid-labile hydrazone is hydrolyzed in the acidic intracellular milieu. Subsequently, an adjacent disulfide bond is reduced by glutathione (GSH), enabling formation of a thiol intermediate that undergoes intramolecular cyclization to ultimately release the active cytotoxic species [77]. Both Mylotarg and Besponsa employ this general linker strategy; however, their plasma stability in practice has been less than anticipated, and the advantages of hydrazone-based designs over other cleavable linkers have not been consistently evident.

#### 5.2.2. Enzyme-Dependent Linkers

##### Peptide Linkers

Protease-cleavable peptide linkers constitute one of the most widely used classes of cleavable linkers in modern ADCs. These linkers rely on lysosomal proteases, most notably cathepsin B (as well as related enzymes such as cathepsin L), to hydrolyze specific peptide bonds and trigger payload release. Cathepsin B is a member of the cysteine protease family; it is enriched in late endosomes and lysosomes of mammalian cells and is frequently overexpressed in multiple cancer types. Accordingly, peptide linkers incorporating defined amino-acid motifs (e.g., Val–Cit or Phe–Lys) are designed to remain stable in systemic circulation yet become efficiently cleaved after internalization and trafficking to lysosomes, thereby liberating the free, bioactive payload.

**Figure 4:**
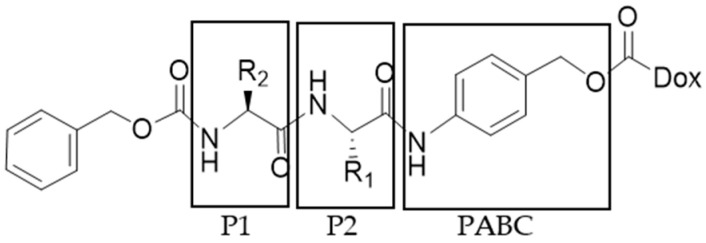
Representative cathepsin-cleavable dipeptide linker design for doxorubicin prodrugs, highlighting the P1 and P2 residues that govern enzymatic recognition and plasma stability, together with the self-immolative PABC spacer.

**Figure 5:**
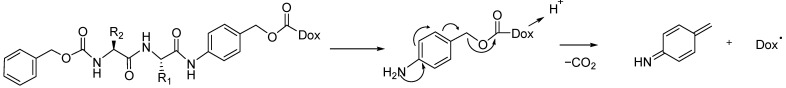
Representative protease-triggered release mechanism of a Val–Cit–PABC-type peptide linker, illustrating dipeptide cleavage followed by self-immolative 1,6-elimination and payload liberation.

Early work using cathepsin B–substrate dipeptides to design doxorubicin prodrugs established key structure–activity relationships (SAR) for these peptide triggers: a hydrophilic residue at the P1 position (e.g., citrulline or arginine) is typically required for efficient enzymatic recognition, whereas incorporation of a hydrophobic residue at P2 (e.g., phenylalanine, valine, or alanine) can markedly improve plasma stability (Figure 4). In later designs, this protease-cleavable dipeptide motif was frequently combined with a self-immolative spacer, most commonly p-aminobenzyl carbamate (PABC/PABA), to facilitate enzyme–substrate engagement and reduce steric hindrance around the payload. Following proteolytic cleavage, this spacer undergoes a spontaneous 1,6-elimination reaction under intracellular conditions, resulting in payload release along with byproducts such as CO_2_ and a reactive quinone methide intermediate. Clinically, Adcetris (brentuximab vedotin) exemplifies this design by employing the Val–Cit–PABC peptide linker, which provides strong plasma stability and reduced off-target toxicity (Figure 5). Such protease-cleavable linkers are compatible with diverse payload classes, including microtubule inhibitors (MMAE/MMAF) and DNA-damaging agents, and their cleavage kinetics can be tuned by optimizing the peptide sequence to match distinct tumor and lysosomal protease environments [78].

##### Phosphatase- and Pyrophosphatase-Responsive Linkers

**Scheme 2:**
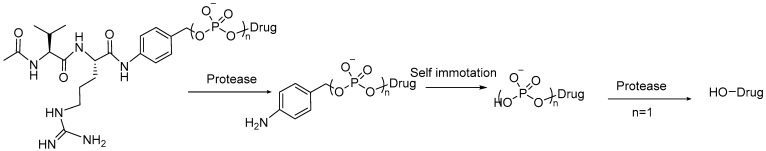
Proposed stepwise activation pathway of a phosphate-containing linker architecture after intracellular processing.

Similar to cathepsins, pyrophosphatases and phosphatases belong to a class of hydrolases that are selectively enriched in lysosomal compartments. Researchers at Merck have reported linker designs incorporating phosphate or pyrophosphate motifs and paired them with the cathepsin B-sensitive Val-Cit-PABA platform. The central goal of this strategy is to enable targeted delivery of glucocorticoids, wherein the phosphate/pyrophosphate group is positioned between the self-immolative spacer (PABA/PABC) and the payload; that is, the phosphate or pyrophosphate moiety is directly linked at the junction between PABA and the effector molecule, thereby introducing an additional lysosome-biased activation step for controlled payload release (Scheme 2).

After tumor cell internalization, payload release from these constructs is proposed to proceed in a stepwise cascade. Specifically, the sequence can involve cathepsin B-mediated cleavage of the Val-Cit trigger, followed by fragmentation of the self-immolative spacer (PABA/PABC), and then phosphatase-catalyzed dephosphorylation when a phosphate motif is present (*n* = 1). For pyrophosphate architectures (*n* = 2), an additional pyrophosphatase-dependent step may be required to complete activation and payload liberation. A key advantage of this linker class lies in its solubility-enhancing features: the hydrophilic, permanently charged phosphate/pyrophosphate groups not only enable efficient bioconjugation of otherwise highly lipophilic glucocorticoid derivatives but also markedly facilitate downstream purification. This, in turn, can reduce residual free linker-related impurities in the final ADC product to below 0.10%. In vitro, ADCs bearing either phosphate or pyrophosphate motifs have demonstrated clear biological activity.

**Scheme 3:**
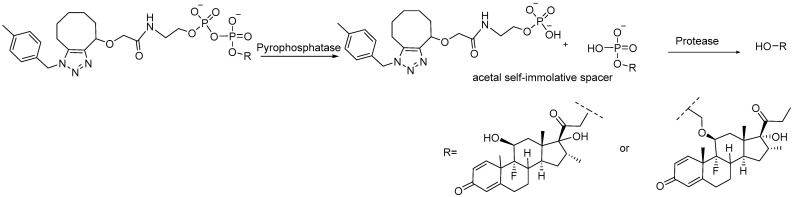
Proposed release pathway of a pyrophosphate-containing linker architecture for hydroxyl-bearing payloads after intracellular processing.

The same Merck research team has also developed a distinctive pyrophosphatase-dependent linker platform designed to enable the targeted release of hydroxyl-containing payloads, such as dexamethasone and fluticasone propionate [79] (Scheme 3). ADCs constructed using this approach exhibited excellent stability in vitro and demonstrated potent cytotoxic activity against tumor cell lines.

##### Glucuronide Linkers

**Figure 6:**
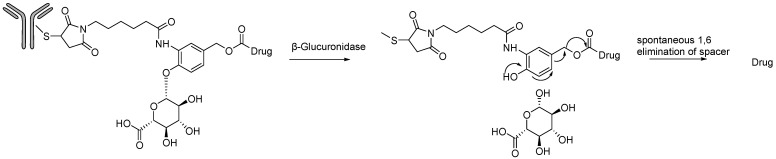
Representative β-glucuronidase-cleavable glucuronide linker architecture used in ADC design.

Among enzyme-responsive cleavable linkers, β-glucuronidase-triggered designs offer a particularly high degree of selectivity. β-Glucuronidase is a member of the glycosidase family and catalyzes the hydrolysis of β-D-glucuronic acid residues. Importantly, this enzyme is enriched not only in lysosomes but also in the tumor stroma/microenvironment. Linkers bearing glucuronide motifs can therefore remain largely inert in normal tissues, where β-glucuronidase expression is typically low, while being preferentially activated in tumors, especially solid tumors, where extracellular matrix remodeling and cell turnover can release substantial amounts of β-glucuronidase into the local milieu (Figure 6). Enzymatic cleavage of the glucuronide bond subsequently enables payload liberation. This linker strategy has been incorporated into multiple ADCs under clinical investigation (e.g., the TROP2-targeting SGN-TROP2X) and has demonstrated plasma stability markedly superior to that of hydrazone linkers, together with strong tumor-selective activation. Consequently, β-glucuronidase-cleavable linkers may reduce normal-tissue injury and are considered well suited for solid tumor applications.

##### β-Galactosidase-Cleavable Linkers

**Figure 7:**
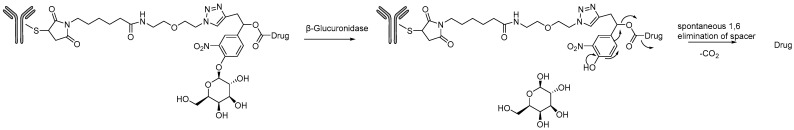
Representative β-galactosidase-cleavable linker incorporating a PEG10 spacer and a nitro-substituted self-immolative motif.

An ADC incorporating a β-galactosidase-cleavable linker has also been reported, in which a PEG10 spacer is embedded within the linker architecture (Figure 7). This spacer was further modified with a nitro substituent to accelerate the subsequent self-immolative fragmentation. Similar to β-glucuronidase-responsive designs, the dissociation mechanism of this linker class is initiated by β-galactosidase-mediated hydrolysis of the galactoside trigger, a process that can endow the chemical precursor with improved hydrophilicity prior to payload release. A key distinction, however, is that β-galactosidase is largely confined to lysosomes, whereas β-glucuronidase is expressed not only in lysosomes but can also be abundant in the solid-tumor microenvironment [80]. Experimental data further indicated that, in an anti-HER2 ADC designed to release MMAE, the β-galactosidase–linker ADC demonstrated superior efficacy compared with T-DM1 in both in vitro cellular assays and in vivo animal models.

## 6. Payloads

Payloads are the principal determinants of the intracellular pharmacological activity of antibody–drug conjugates (ADCs). As cytotoxic agents covalently attached to the antibody through a linker, payload properties are critically important because their mechanisms of action govern antitumor potency and, consequently, influence the therapeutic index and clinical applicability of ADCs. To date, the payloads used in approved ADCs are uniformly highly potent, with the major classes including auristatins (MMAE/MMAF), calicheamicins, maytansinoids (DM1/DM4), and topoisomerase I inhibitors such as SN-38 and deruxtecan (DXd). These payloads are generally far more potent than conventional chemotherapeutics, in some cases exhibiting activity in the picomolar range. Notably, payloads used in approved ADCs predominantly target microtubules or DNA/DNA-associated processes [35]. Below, we summarize the payloads used in marketed ADCs.

### 6.1. Microtubule-Disrupting Agents

Tubulin is the fundamental building block of microtubules. Microtubule-targeting agents bind to tubulin and disrupt the dynamic polymerization-depolymerization behavior of microtubules, leading to mitotic arrest at the G2/M phase and ultimately triggering apoptosis [8,35,81,82,83,84]. In ADC development, microtubule-directed payloads are primarily derived from four major classes: auristatin derivatives (e.g., MMAE and MMAF), maytansinoids (e.g., DM1 and DM4), tubulysins, and halichondrin-derived agents (e.g., eribulin).

#### 6.1.1. Auristatins

**Figure 8:**
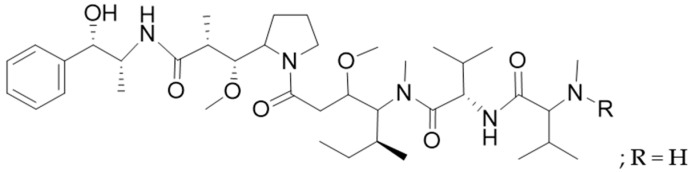
Structure of monomethyl auristatin E (MMAE).

Dolastatin-like bioactive peptides, originally isolated from the Indian Ocean mollusk “Dolabella auricularia”, exhibit potent antimitotic activity and marked growth-inhibitory effects against a wide range of cancer cell types. Based on this structural scaffold, a series of synthetic derivatives with improved pharmaceutical properties, collectively termed auristatins, has been developed. Auristatins act by binding to tubulin and perturbing the conformation of the tubulin heterodimer, thereby disrupting microtubule dynamics and suppressing microtubule assembly/disassembly through interference with tubulin-dependent GTP hydrolysis [85,86,87]. Auristatins are among the most widely used payload classes in ADC development; notably, monomethyl auristatin E (MMAE) has been successfully incorporated into the marketed ADCs Adcetris and Polivy (Figure 8). In addition, numerous clinical-stage ADC candidates employ auristatin-based payloads such as MMAE or monomethyl auristatin F (MMAF).

The structure–activity relationships (SAR) of this payload class have been investigated in considerable depth, with efforts focusing largely on modifications at the terminal subunits (i.e., the N-terminus and C-terminus) [87]. Among these approaches, installation of a carbamate functionality at the N-terminus has emerged as one of the most commonly employed structural optimization strategies.

**Figure 9:**
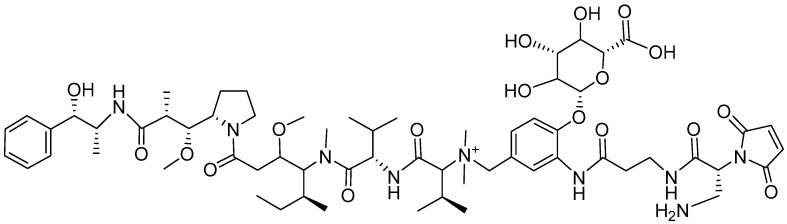
Seattle Genetics’ linker–payload design incorporating an N,N-dimethyl auristatin derivative and a quaternary ammonium linkage.

In 2015, a research team at Seattle Genetics expanded the chemical space of ADC payloads to include tertiary amine-containing compounds, with a particular emphasis on N,N-dimethyl auristatin derivatives (Figure 9). Notably, the group reported one of the first examples of antibody conjugation through a quaternary ammonium linkage, enabling covalent attachment of the payload to a monoclonal antibody. The resulting ADCs exhibited good stability under physiological conditions and demonstrated high potency in vitro and in vivo, together with strong target-dependent specificity. This work further broadened the repertoire of payload chemotypes and conjugation strategies available for targeted drug delivery via ADCs.

**Figure 10:**
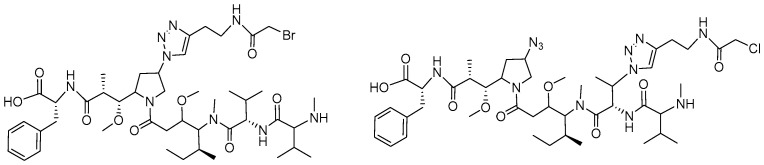
The structure of azide (-N_3_) auristatin derivatives: Novel hydrophilic derivatives.

More recently, Agensys reported an alternative optimization strategy in which an azide (-N_3_) functionality was introduced into the central subunit of the scaffold. Following conjugation of this modified chemotype to a protease-cleavable linker, the resulting constructs yielded more hydrophilic derivatives (Figure 10), with improved potency observed in both in vitro and in vivo settings. This approach provides an additional technical route for linker–payload conjugation and expands the toolbox for tuning physicochemical properties during ADC design.

In auristatin derivatives that contain both an amine and an alcoholic hydroxyl as potential conjugation handles, linker attachment is typically prioritized through formation of a carbamate linkage at the amine. Seattle Genetics subsequently introduced an alternative conjugation strategy that enables antibody attachment of hydroxyl-bearing payloads using a methylene alkoxy carbamate (MAC) motif. By incorporating both a basic substituent and an electron-withdrawing group in proximity to the carbamate nitrogen, the resulting conjugates maintain physiological stability while exhibiting high potency and pronounced target-dependent specificity in both in vitro and in vivo settings.

**Figure 11:**
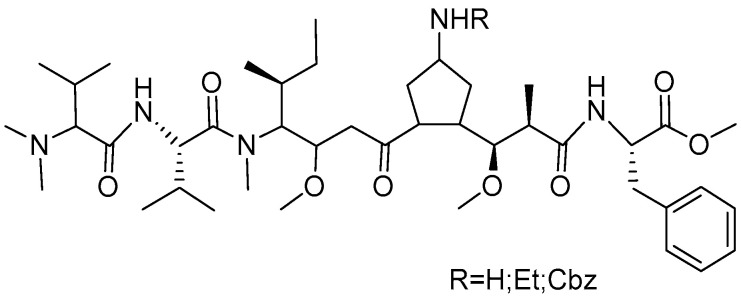
The structure of azastatins.

In addition, researchers at Uppsala University have developed a new class of highly potent auristatin derivatives (Figure 11). These molecules feature an antibody-attachment handle located on the central amine side chain, providing a defined conjugation site within the scaffold. Available studies indicate that this new class of highly potent auristatin derivatives represents promising next-generation cytotoxic payloads suitable for ADC development.

#### 6.1.2. Maytansinoid Derivatives (DM1, DM4)

**Figure 12:**
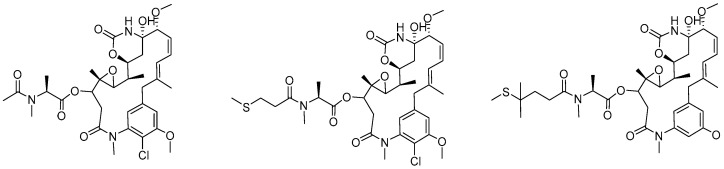
Structures of Maytansine, DM1 and DM4.

Maytansinoids exert their antimitotic effects primarily by disrupting microtubule assembly, thereby preventing the polymerization of tubulin dimers and inhibiting the formation of mature microtubules. They display extraordinarily potent antiproliferative activity, reflecting strong cytotoxic potential. However, the parent natural product lacks suitably reactive functional groups, making direct antibody conjugation challenging. To overcome this limitation, researchers developed a series of highly potent thiomethyl (SMe)-containing derivatives, among which DM1 and DM4 are prototypical examples (Figure 12).

**Figure 13:**
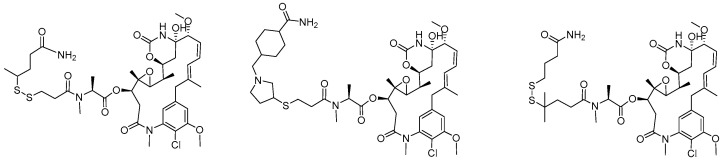
Representative linker attachment modes of DM1 and DM4, illustrating disulfide-type and sulfide/thioether-type conjugation patterns.

DM1 and DM4 are commonly conjugated to linkers via disulfide bonds. Such disulfide-based linkages can provide adequate stability in systemic circulation, while remaining reducible within the intracellular environment, thereby enabling efficient cleavage and release of the active payload inside target cells (Figure 13).

**Figure 14:**
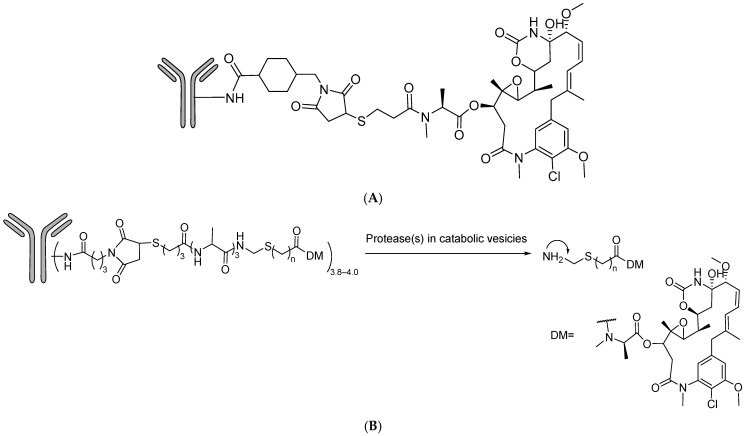
(**A**) Representative maytansine-derived drug–linker precursor showing the secondary hydroxyl group used for linker installation. (**B**) Structure of the new ADCs bearing peptide-immolative linkers and the metabolites that are expected to be formed in catabolic vesicles followed by S-methylation.

In addition, several maytansinoid-based ADCs utilize a secondary hydroxyl group as the conjugation handle, and many of these constructs employ transglutaminase-mediated bioconjugation linkers. For example, an ADC derived from daratumumab has been shown to selectively deliver DM4 to cancer cells with high CD38 expression. More recently, ImmunoGen reported a next-generation ADC featuring a sulfur-containing maytansine analog payload conjugated to the antibody via a highly stable tripeptide linker, again using the same secondary hydroxyl attachment site (Figure 14). Compared with earlier maytansinoid ADC designs, increasing the number of methylene units within this linker architecture enhanced bystander killing and improved therapeutic efficacy in murine models. To improve clarity, Figure 14 explicitly indicates the secondary hydroxyl group used for linker installation, thereby showing the point at which the payload is converted into a linker-compatible precursor.

**Figure 15:**
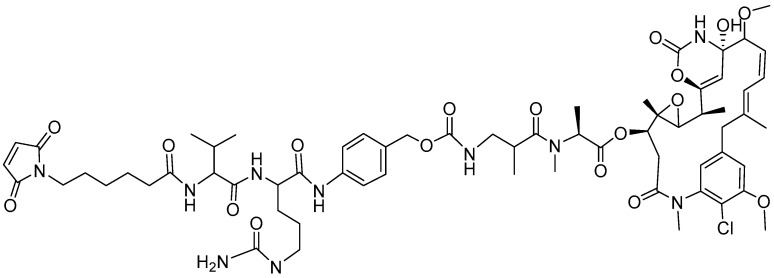
Representative maytansinoid derivative generated by macrocycle modification and amine-based linker derivatization.

Using a similar design rationale, researchers at Regeneron and Abzena explored a series of structural modifications while preserving the maytansinoid macrocyclic core. These efforts evaluated the impact of N-substitution at the N-methylalanine nitrogen, fine-tuning of macrocycle side-chain length, and conjugation via primary- or secondary-amine-based linkers on pharmacological activity and overall payload performance (Figure 15).

#### 6.1.3. Tubulysins

**Figure 16:**
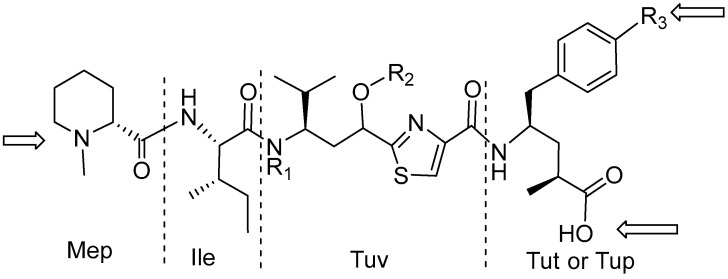
Representative tubulysin scaffold showing the Tut/Tup module and its linker-compatible conjugation handle.

Tubulysins are potent inhibitors of microtubule polymerization that induce rapid collapse of the cytoskeleton in dividing cells, thereby triggering apoptosis. They are naturally occurring tetrapeptides composed of Mep, Ile, Tuv and either Tut (R_3_ = OH) or Tup (R_3_ = H) units (Figure 16).

**Figure 17:**
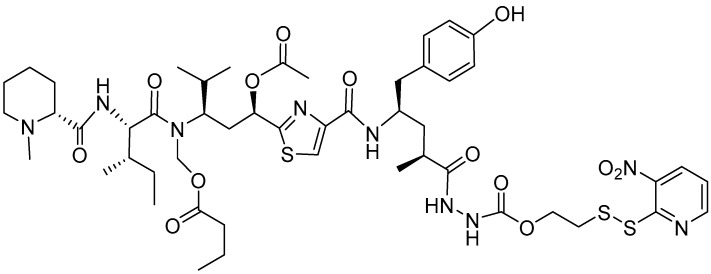
Representative tubulysin-derived conjugation strategy centered on the Tut/Tup carboxylic acid moiety, showing hydrazide coupling and the position of the cleavable linker segment in an EC1428-like construct.

In ADC development, multiple potential conjugation handles on tubulysin scaffolds have been extensively explored. A well-defined attachment site is the carboxylic acid functionality within the Tut or Tup module. For example, Endocyte’s EC1428 was constructed by coupling this carboxyl group to a linker via a hydrazide moiety (Figure 17). Using a similar derivatization strategy, Oncomatryx introduced a cleavable maleimide linker incorporating a PABA-Val-Cit motif at the same position to enable controlled intracellular payload release.

**Figure 18:**
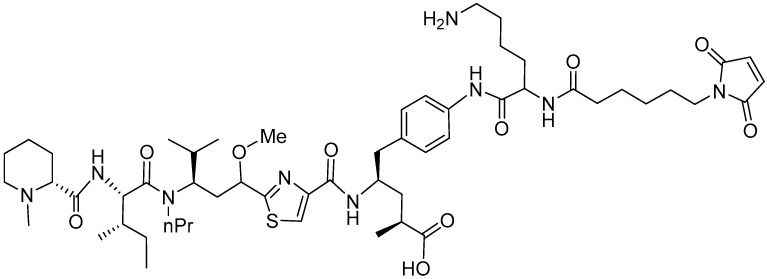
Examples of Tubulysin-derived payload linked through a modified phenyl group.

AstraZeneca, Bristol Myers Squibb, and Pfizer have pursued an alternative modification route in which linker attachment is enabled through derivatization of the phenyl ring within Tup/Tut motifs, thereby creating suitable conjugation handles without disrupting the pharmacophore (Figure 18). In parallel, extensive work has also validated linker conjugation strategies involving the Mep substituent. Notably, researchers at Ingenica reported that desmethyl-Mep analogues can retain potent cytotoxicity and thus represent highly attractive ADC payload candidates; importantly, the secondary amine on this scaffold can be functionalized with a non-cleavable maleimide–hexyl linker, enabling robust antibody conjugation.

Oncomatryx further demonstrated that, when the Mep moiety is replaced by alternative motifs bearing a secondary amine, installation of a cleavable linker via a carbamate bond provides an effective route for ADC construction. Notably, Genentech has reported a distinct strategy in which a quaternary ammonium handle is used to couple linkers to tertiary amine–containing payloads. Introduction of an Mc-val–cit–PABA linker into such payloads markedly increases conjugate hydrophilicity and can improve stability in systemic circulation. Seattle Genetics adopted a conceptually related approach by employing glucuronide-based linkers to enhance hydrophilicity and to exploit the elevated β-glucuronidase activity in cancer cells, thereby enabling selective intracellular cleavage of the payload.

**Figure 19:**
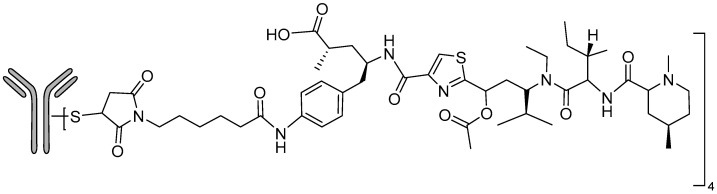
Tubulysin-derived payload structure (AZ13599185) and its incorporation into the bispecific HER2-targeting ADC MEDI4276.

In addition, AstraZeneca/MedImmune developed the bispecific ADC MEDI4276, which targets two distinct epitopes on HER2. Bispecific engagement may enhance receptor clustering and increase internalization, potentially augmenting intracellular payload release and tumor cell killing. MEDI4276 consists of a bispecific anti-HER2 antibody conjugated to the tubulysin-derived payload AZ13599185 through a non-cleavable linker, with a DAR of 4 (Figure 19). In preclinical studies, MEDI4276 showed robust antitumor activity, whereas hepatotoxicity emerged as a principal dose-limiting toxicity in clinical evaluation. In Figure 16, the linker-bearing payload is shown in relation to the schematic antibody so that the site of attachment and the overall architecture of MEDI4276 can be more readily appreciated.

#### 6.1.4. Eribulin

**Figure 20:**
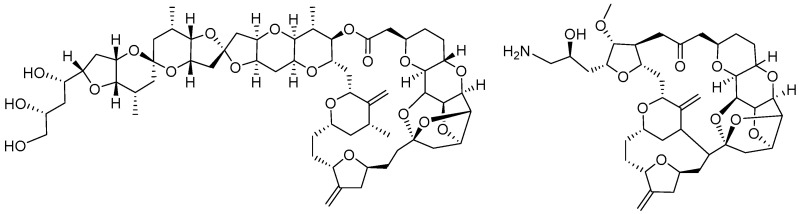
Structures of Halichondrin B and Eribulin.

Eribulin is a fully synthetic analogue derived from halichondrin B, a macrocyclic natural product originally isolated from marine sponges (Figure 20). It selectively targets the (+) end of β-tubulin at microtubule plus ends, primarily by inhibiting microtubule polymerization/elongation while exerting minimal effects on microtubule depolymerization. This distinctive mechanism of action underpins its clinical activity in certain settings of taxane resistance, allowing therapeutic benefit to be retained in patients who are refractory to paclitaxel. In November 2010, the U.S. Food and Drug Administration (FDA) approved eribulin for the treatment of metastatic breast cancer. ADCs incorporating eribulin as a cytotoxic payload have attracted interest because they may confer a pronounced bystander effect, supporting potential clinical utility in heterogeneous tumors. In addition, eribulin shows relatively low sensitivity to P-glycoprotein (P-gp)-mediated efflux, which may further broaden its applicability in drug-resistant disease contexts.

**Figure 21:**
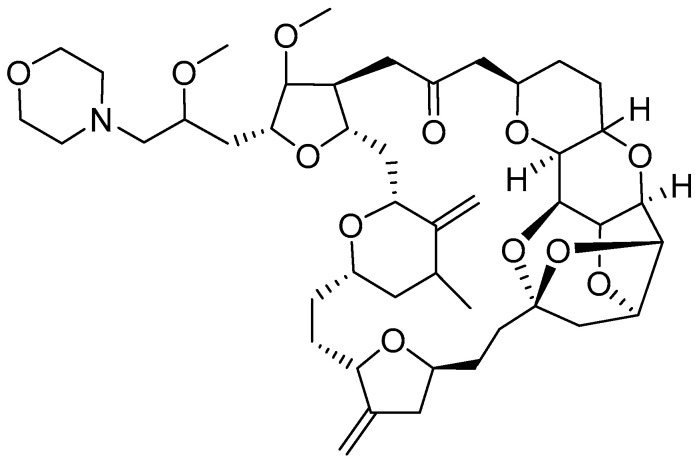
Structures of representative eribulin derivatives.

Earl F. Albone and colleagues designed an eribulin-based ADC by installing a linker at the C-35 primary amine of the payload. The resulting conjugate showed pronounced antiproliferative activity against the ovarian cancer cell line IGROV1 (IC_50_ ≈ 20 pmol/L). In a non-small cell lung cancer (NSCLC) NCI-H2110 xenograft model, administration of the ADC at 5 mg/kg produced complete tumor eradication, supporting the feasibility of eribulin as a microtubule-targeting payload for ADC development. Building on this concept, medicinal-chemistry optimization of the C32 side chain, either by attenuating the basicity of amine-containing fragments or by increasing overall lipophilicity, yielded Eribulin derivatives, which demonstrated broad in vitro and in vivo antitumor activity across multiple xenograft models (Figure 21). Importantly, these modifications were pursued to improve oral bioavailability, enhance brain penetration (including increased cerebrospinal fluid exposure), and reduce susceptibility to P-glycoprotein (P-gp)-mediated efflux, suggesting potential utility not only in systemic malignancies but also in indications such as brain tumors.

**Figure 22:**
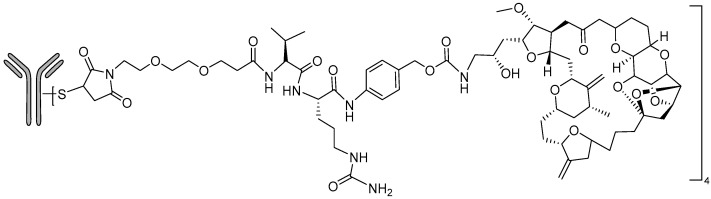
Structure of the FRα-targeting eribulin-based ADC MORAb-202.

In the development landscape of folate receptor-α (FRα)-targeting ADCs, Eisai’s farletuzumab ecteribulin (MORAb-202) incorporates the humanized anti-FRα monoclonal antibody farletuzumab and uses eribulin as the cytotoxic payload (Figure 22). The conjugate is assembled via a cathepsin B-cleavable peptide linker (Val-Cit) with a self-immolative spacer, and has been reported to employ an average drug-to-antibody ratio (DAR) of 4.

Among FRα-targeting programs, ImmunoGen’s mirvetuximab soravtansine has been one of the most clinically mature, and the Phase III forward I trial reported a confirmed overall response rate (ORR) of 22% in the overall study population. In a first-in-human Phase I study of MORAb-202 (NCT03386942, *n* = 22), MORAb-202 demonstrated encouraging activity with an ORR of 45.5% (10/22), including one complete response and nine partial responses, together with a generally manageable safety profile. Notably, grade 1/2 interstitial lung disease (ILD)/pneumonitis considered related to MORAb-202 was identified in 5 patients (23%) following independent adjudication.

#### 6.1.5. Cryptomycins

**Figure 23:**
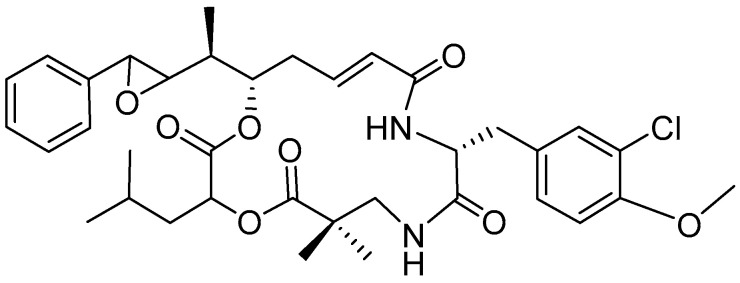
Structure of Cryptomycin-1.

Cryptomycins are macrocyclic hexadepsipeptides with potent antitumor activity (Figure 23). However, early clinical trials showed that doses required for therapeutic efficacy were associated with unacceptable toxicity.

**Figure 24:**
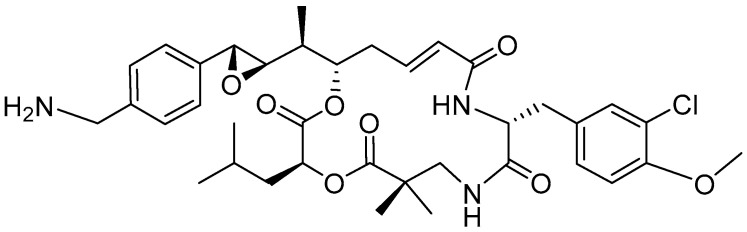
Structure of Cryptophycin benzylamine analog.

Several groups have attempted to develop cryptomycins as ADC payloads, but these molecules lack obvious native conjugation sites. Two main strategies have been employed to introduce linker attachment functionalities. First, Genentech converted an aromatic ring in the cryptomycin scaffold into a benzylamine, yielding a highly potent payload suitable for carbamate-based conjugation (Figure 24).

**Figure 25:**
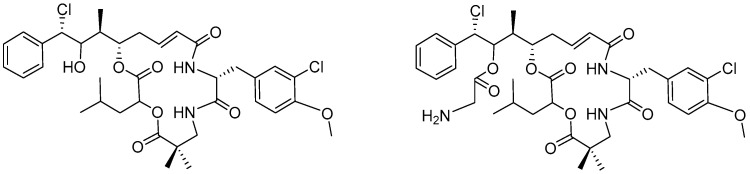
Structures of Cryptomycin-55 and Cryptomycin-52.

Second, researchers at Sichuan University exploited CR55, a prodrug form of cryptomycin-52 (CR52), which undergoes ring closure under physiological conditions to regenerate active CR52; this transformation provides an opportunity to install linker attachment sites (Figure 25).

#### 6.1.6. Antimitotic Eg5 Inhibitors

**Figure 26:**
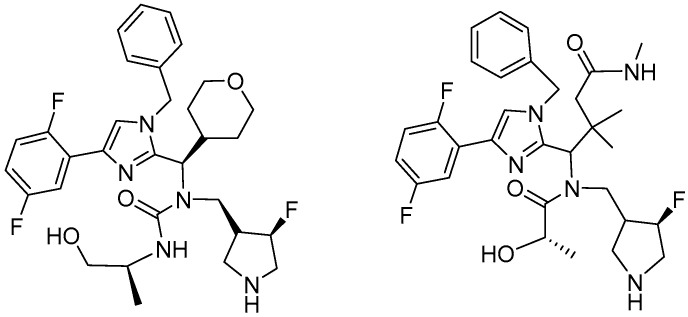
Structures of Eg5 inhibitors.

The kinesin spindle protein (KSP), also known as Eg5 or KIF11, is an ATP-dependent motor protein required for centrosome separation during mitosis. Inhibiting Eg5 disrupts spindle assembly and arrests cells in mitosis, making KSP inhibitors an attractive strategy for anticancer therapy. Kinesin spindle protein (KSP, also known as Eg5 or KIF11) is an ATP-dependent motor protein that participates in centrosome separation during cell-cycle progression. Eg5 is highly expressed in both hematologic malignancies, such as acute myeloid leukemia (AML) cells and diffuse large B-cell lymphoma (DLBCL), and solid tumors, including breast, bladder, and pancreatic cancers. This expression pattern is closely associated with poor clinical outcomes, making Eg5 a highly attractive target for cancer therapy. Eg5 inhibitors developed by Novartis have been employed as ADC payloads and, upon conjugation to antibodies via non-cleavable linkers, have been used to target HER2-expressing cells (Figure 26).

**Figure 27:**
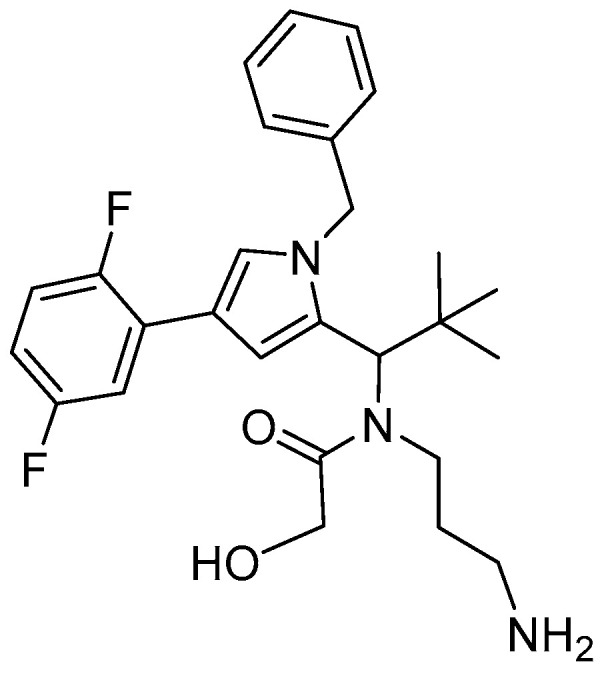
Structure of an Eg5 pyrrole-subseries inhibitor used as an ADC payload.

Anette Sommer and colleagues employed a non-cell-permeable Eg5 pyridine inhibitor as the payload to construct a novel IL3RA-targeted ADC, which exhibited potent and highly selective antiproliferative activity. Carsten Terjung further evaluated the feasibility of using a new Eg5 pyrrole-subseries inhibitor as an ADC payload (Figure 27). In urothelial cell carcinoma (UCC) xenograft models, ADCs incorporating this inhibitor demonstrated excellent antitumor efficacy, achieving complete tumor eradication and outperforming ado-trastuzumab emtansine (Kadcyla) in therapeutic effect.

### 6.2. DNA-Damaging Agents

Compared with microtubule inhibitors, DNA-directed cytotoxic agents can damage genomic DNA through mechanisms such as double-strand breaks, alkylation, intercalation, and crosslinking, thereby exerting cytotoxic effects across the entire cell cycle and often demonstrating strong activity in solid tumors. Because the number of functional DNA targets per cell is limited relative to the abundant and dynamic microtubule network, an equivalent intracellular payload load can translate into more efficient tumor cell killing for DNA-targeting warheads. Moreover, ADCs carrying DNA-active payloads may retain efficacy in tumors with lower antigen density, which is one reason why many next-generation ADC programs have increasingly adopted DNA-targeting payload classes.

DNA-targeting payloads can be broadly categorized into DNA-damaging agents and topoisomerase inhibitors. The DNA-damaging class includes key modalities such as calicheamicins, pyrrolobenzodiazepines (PBDs), and duocarmycins. In contrast, topoisomerase I inhibitors are predominantly represented by camptothecin-derived compounds, including exatecan, SN-38, and deruxtecan (DXd) [88,89,90].

#### 6.2.1. DNA Cross-Linkers: PBD and IBD Dimers

Pyrrolo [2,1-c] benzodiazepines (PBDs) are a class of antitumor natural products that exert cytotoxicity by selectively binding within the DNA minor groove. Their key mechanism involves covalent DNA alkylation: the electrophilic N10/C11 imine of the PBD scaffold reacts with the exocyclic N2 of guanine to form a covalent adduct, thereby modifying DNA and disrupting essential cellular processes [91].

**Figure 28:**
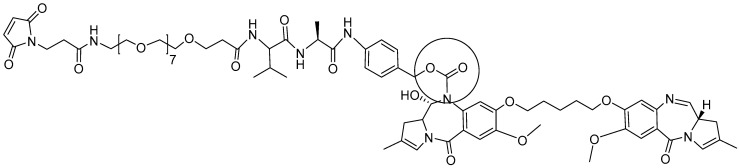
Attachment of linkers to PBD payload.

Seattle Genetics utilized the aniline functionality of SGD1882 as a conjugation handle. This site is conceptually analogous to the p-aminobenzyl (PAB) motif commonly used in cleavable linker systems, enabling intracellular processing that can ultimately release the free PBD payload. In parallel, Stemcentrx and Spirogen developed PBD-ADC constructs by coupling the payload through the N10 position of the PBD via a carbamate linker (Figure 28).

**Figure 29:**
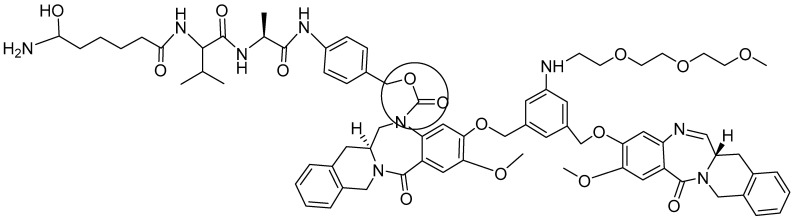
Attachment of linkers to IBD payloads.

ImmunoGen applied the same carbamate-based strategy to structurally related indolinobenzodiazepine (IBD) dimers and also developed an alternative design in which a substituted phenyl ring serves as a bridge connecting two IBD monomers at their C8/C8’ positions (Figure 29).

**Figure 30:**
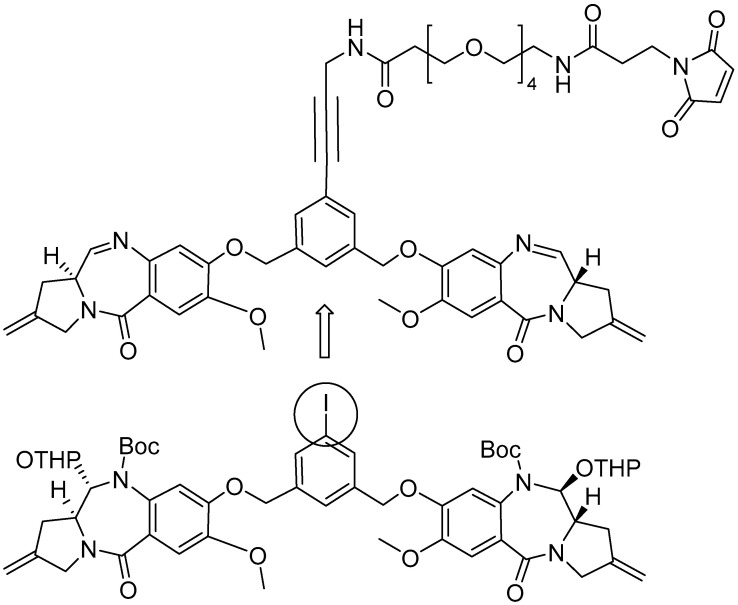
PBD dimer/linker attachment using a common iodobenzene intermediate.

In a similar vein, Spirogen and Genentech designed PBD derivatives linked through an iodobenzene bridge (Figure 30). Transition metal-catalyzed reactions such as Sonogashira coupling, Buchwald–Hartwig amination, and azide-alkyne “click” chemistry were used to introduce alkyne-, piperazine- or triazole-containing linkers, respectively, to generate a variety of linker–payload conjugates.

#### 6.2.2. DNA Double-Strand Break Inducers: Calicheamicins

**Figure 31:**
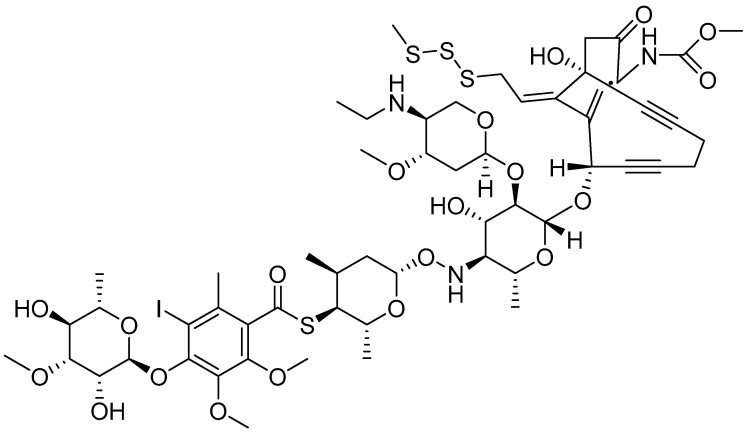
Representative calicheamicin payload structure used in ADC design.

Calicheamicins are among the most extensively studied enediyne antibiotics, their distinctive structural features and mechanistically complex DNA-damaging activity have established them as a major payload class in ADC research (Figure 31). Calicheamicin-based conjugation strategies have been clinically validated through their incorporation into the marketed ADCs Mylotarg and Besponsa.

**Figure 32:**
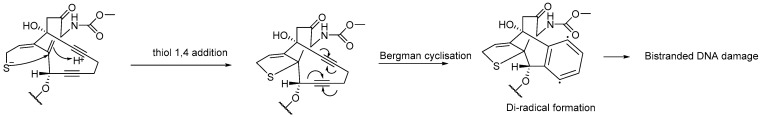
Proposed diradical-generating activation mechanism of calicheamicin following intracellular trigger release.

Intracellular payload release from calicheamicin-based ADCs proceeds via a two-step process (Figure 32). First, the acid-sensitive hydrazone bond is cleaved in the acidic intracellular compartment. Second, the disulfide bond is reduced by intracellular GSH, generating a thiol that triggers intramolecular 1,4-addition to an enone, initiating a Bergman cyclization reaction. This cyclization produces a highly reactive diradical intermediate, which abstracts hydrogen atoms from the deoxyribose backbone, causing DNA double-strand breaks and ultimately leading to cell death.

**Figure 33:**
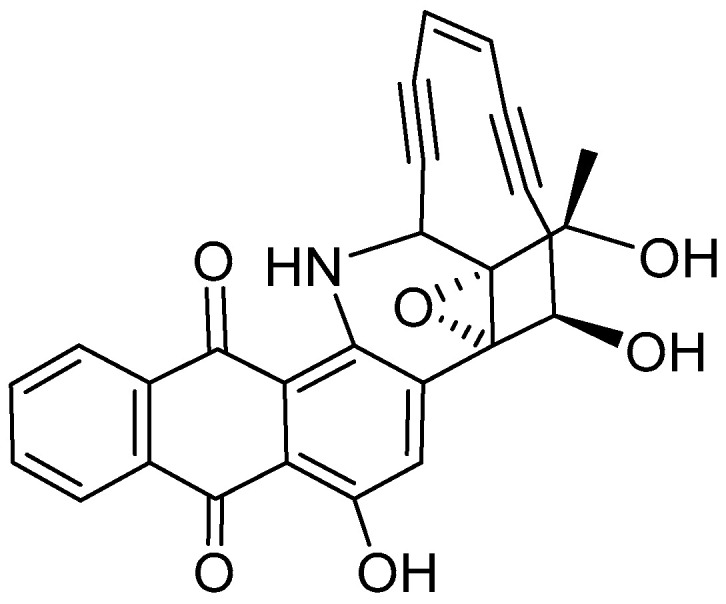
Representative uncialamycin payload structure and its derivatization potential for ADC conjugation.

Recently, a novel enediyne natural product, uncialamycin, was isolated from a Streptomyces species associated with lichen in British Columbia (Figure 33). Its structure has been confirmed through total synthesis, and multiple highly potent synthetic analogs have since been developed as potential ADC payloads.

Research at Bristol Myers Squibb (BMS) showed that the secondary amine of uncialamycin is poorly reactive under various peptide coupling conditions and is therefore unsuitable as a conjugation site. Aniline derivatives prepared by direct amination of the aromatic ring still did not provide sufficient reactivity for efficient linker coupling. In contrast, extending the scaffold with an aminoethyl chain to introduce an aliphatic amine provided suitable attachment points for conjugation. Using this payload framework, investigators prepared corresponding prodrug/ADC constructs employing either a protease-cleavable dipeptide linker or a non-cleavable linker. Notably, the cleavable-linker CD70–ADC exhibited highly target-specific cytotoxicity against a renal cancer cell line, whereas the matched non-cleavable construct showed little to no measurable activity in the same model.

**Figure 34:**
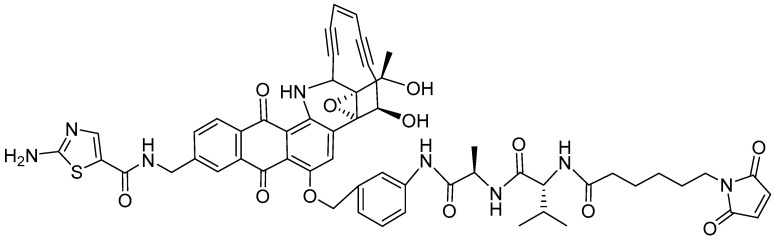
Structures of uncialamycin analogues bearing linker-compatible conjugation sites.

In subsequent work, BMS designed highly potent and chemically stable uncialamycin analogs in which a phenolic group serves as the conjugation site (Figure 34). Using a novel phenolic alkylation strategy, classical cleavable linkers were installed at this position. After antibody conjugation, the resulting ADCs displayed strong antigen-dependent antitumor activity in both in vitro and in vivo studies.

#### 6.2.3. DNA Alkylators: Duocarmycins

**Figure 35:**
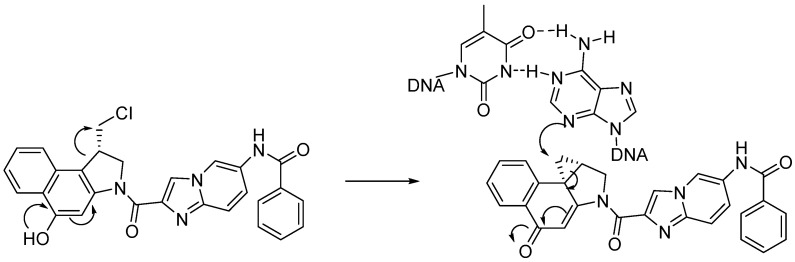
Activation pathway of duocarmycin-type prodrugs, showing phenol-triggered intramolecular cyclization to generate the electrophilic cyclopropane warhead and subsequent alkylation of adenine N3 in the DNA minor groove.

Duocarmycins are highly potent cytotoxic agents that bind within the DNA minor groove and mediate alkylation at the N_3_ position of adenine via a highly reactive cyclopropane motif (Figure 35) [92]. In contrast, the non-cyclized halomethyl precursors of duocarmycins display markedly reduced cytotoxicity; their activation depends on an intramolecular triggering process in which a phenolic group serves as an internal activator to promote cyclization and generate the electrophilic cyclopropane warhead. Consequently, in duocarmycin–ADC development, linker attachment through the phenolic functionality is a central design strategy, as it enables controlled activation and payload release at the target site.

**Figure 36:**
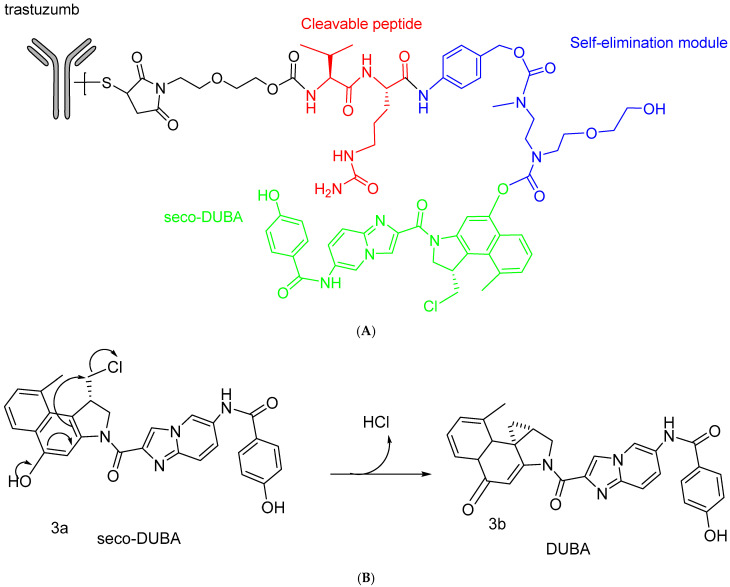
(**A**) Structure of the duocarmycin-based ADC construct developed by Synthon (SYD985); (**B**) schematic representation of the Winstein spirocyclization of seco-duocarmycin 3a (seco-DUBA) to its spiro analogue 3b (DUBA).

In Synthon’s SYD985, the phenolic group is coupled via a bis-carbamate linkage to an Mc-val-cit-PABC-based duocarmycin prodrug payload [92,93,94] (Figure 36A). Ronald C. Elgersma set out to develop a new class of linkerdrugs based on duocarmycins, potent DNA-alkylating agents that are composed of a DNA-alkylating and a DNA-binding moiety and that bind into the minor groove of DNA. Linkerdrugs were evaluated as ADCs by conjugation to the antiHER2 antibody trastuzumab via reduced interchain disulfides. Duocarmycin 3b, bearing an imidazo[1,2-a]pyridine-based DNA-binding unit, was selected as the drug moiety, notably because of its rapid degradation in plasma. The drug was incorporated into the linker-drugs in its inactive prodrug form, secoduocarmycin 3a. Linker attachment to the hydroxyl group in the DNA-alkylating moiety was favored over linking to the DNAbinding moiety. Following cathepsin B-mediated cleavage of the peptide trigger, liberation of the free phenol promotes an intramolecular rearrangement/cyclization that generates the electrophilic cyclopropane active species (Figure 36B).

#### 6.2.4. Topoisomerase I Inhibitors: Camptothecin

**Figure 37:**
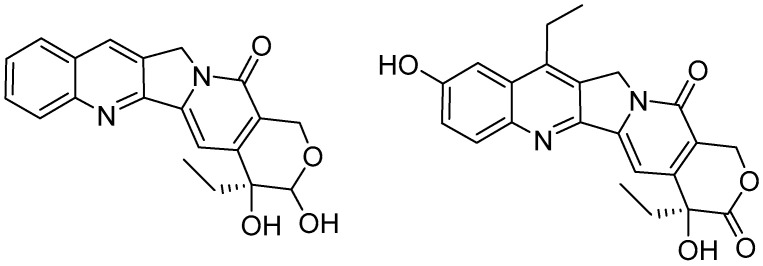
Structures of camptothecin (CPT) and SN-38.

Camptothecin (CPT) and its derivatives are the prototypical topoisomerase I (TOP1) inhibitors. They act by stabilizing the TOP1-mediated DNA single-strand break intermediate, thereby forming a ternary DNA-TOP1-inhibitor complex. When this stabilized complex is encountered by a replication fork, it can be converted into a DNA double-strand break, leading to cytotoxicity. Native CPT is a pentacyclic natural product whose extremely poor aqueous solubility has limited its broader clinical use. In contrast, the water-soluble prodrug irinotecan has been approved for the treatment of metastatic colorectal cancer. SN-38, generated from irinotecan via hepatic carboxylesterase-mediated metabolism, is the principal active metabolite; however, SN-38 can undergo lactone ring opening, which results in an inactive carboxylate form (Figure 37).

Immunomedics established two principal conjugation strategies for coupling SN-38 to antibodies. In one approach, the linker is attached through the more reactive C-10 phenolic hydroxyl, forming a chemically stable carbamate linkage. In the second strategy, conjugation is performed at the C-20 hydroxyl, a position considered critical for in vivo activity, while simultaneously helping to stabilize the lactone ring, thereby preserving the active camptothecin pharmacophore.

**Figure 38:**
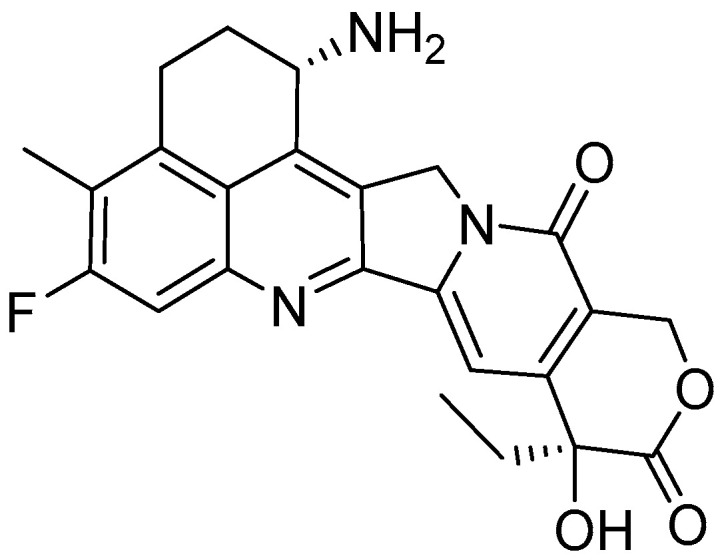
Structure of the exatecan derivative DX-8951f.

Exatecan (DX-8951f) represents another highly potent TOP1-inhibitory chemotype suitable for ADC payload development (Figure 38). As a camptothecin analogue, exatecan features an amine substituent bridging the 7- and 9-positions on the cyclohexyl ring. This amino group improves aqueous solubility, while the conformational rigidity imparted by the cyclohexyl ring helps maintain a favorable equilibrium between the active lactone and the inactive hydrolyzed carboxylate forms. Further derivatization of the amino/hydroxyl functionalities yields deruxtecan (DXd) analogues. Both derivatives retain the biological activity characteristic of the exatecan scaffold.

The free hydroxyl and amino groups on the exatecan scaffold provide well-defined conjugation handles and can be coupled to the payload via an enzyme-cleavable tetrapeptide linker, such as Gly-Gly-Phe-Gly. Upon conjugation to an anti-HER2 antibody, the resulting ADC has demonstrated substantial therapeutic promise against HER2-positive malignancies in clinical evaluation.

Although the cyclohexylamine ring in DXd contributes to stabilization of the bioactive lactone form, the associated stereogenic center(s) increase synthetic complexity and complicate structure–activity relationship (SAR) investigations. To address this limitation, researchers at ImmunoGen developed a series of next-generation CPT analogues in which the cyclohexylamine ring was opened to eliminate the additional chiral center, thereby improving suitability for monoclonal antibody conjugation. When conjugated to an anti–human epidermal growth factor receptor (HuEGFR) antibody, the resulting ADCs showed marked efficacy in an EGFR-positive HSC-2 xenograft model.

#### 6.2.5. Topoisomerase II Inhibitors: Doxorubicin and PNU-159682

Osteosarcoma is the most common malignant bone tumor in children and adolescents, with 40–50% of patients experiencing recurrence or distant metastasis after surgery, leading to a 5-year survival rate below 30%. Doxorubicin, an anthracycline, remains one of the first-line chemotherapeutics for osteosarcoma. It intercalates into the DNA double helix, prevents strand separation and thereby interferes with DNA replication and RNA synthesis. However, when used as an ADC payload, the intrinsic potency of doxorubicin is often insufficient, prompting the development of more potent anthracycline derivatives.

**Scheme 4:**
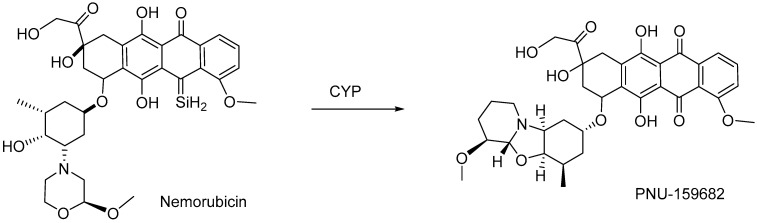
CYP-mediated metabolic conversion of nemorubicin into the active metabolite PNU-159682.

PNU-159682, a hepatic metabolite of nemorubicin, is a Topo II inhibitor with cytotoxicity approximately 100-fold greater than that of doxorubicin. Its biological potency is roughly three orders of magnitude higher than that of nemorubicin, with IC_50_ values in the range of 20–100 pM, and it is not a substrate of common efflux transporters. The biotransformation from nemorubicin to PNU-159682 has been identified as a key metabolic activation pathway in the liver (Scheme 4).

**Figure 39:**
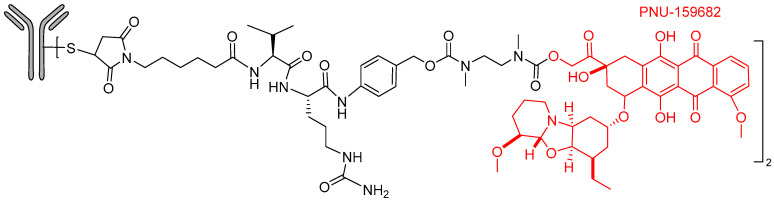
Structure of the PNU-159682-based ADC NMS249.

Genentech developed a novel anti-CD22 ADC, NMS249, based on PNU-159682 as the payload. This ADC employs an MC-VC-PAB-DEA linker and has a DAR of 2 (Figure 39). In xenograft models, the antitumor activity of anti-CD22-NMS249 was comparable to or greater than that of an anti-CD22-VC-MMAE ADC, and it retained efficacy in cell line models that had acquired resistance to CD22-VC-MMAE ADCs. To date, no clinical toxicity data specifically associated with PNU-159682-based ADCs have been reported.

### 6.3. Emerging Payloads

In addition to the conventional payloads described above, numerous non-classical payloads have been explored for ADC development, including inhibitors of anti-apoptotic proteins, splicing modulators, RNA polymerase II inhibitors, proteasome inhibitors and nicotinamide phosphoribosyltransferase (NAMPT) inhibitors.

#### 6.3.1. Inhibitors of Anti-Apoptotic Proteins (Bcl-xL Inhibitors)

**Figure 40:**
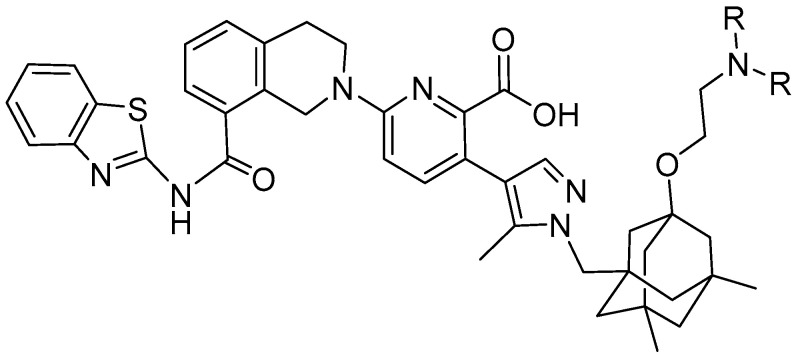
The structure of Bcl-xL inhibitor.

Aberrant overexpression of anti-apoptotic Bcl-2 family proteins, including Bcl-xL, is a major mechanism by which cancer cells acquire resistance to apoptosis. Small molecules that block the BH3-binding groove of Bcl-xL can effectively reactivate apoptotic signaling and trigger tumor cell death [95]. In 2017, AbbVie reported one of the first ADC designs employing a Bcl-xL inhibitor as the payload, enabling targeted delivery to EGFR-positive cells or tissues (Figure 40). To support conjugation, the investigators introduced three distinct attachment sites on the payload scaffold for incorporation of cleavable linkers, and further used aminoalkyl chain extension of the core structure to flexibly install conjugation handles as needed for ADC construction.

#### 6.3.2. Splicing Modulators: Thailanstatin A and Analogs

**Figure 41:**
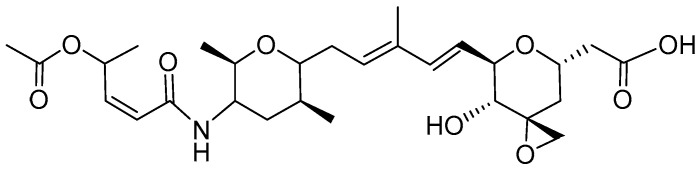
The structure of thailanstatin A.

The spliceosome is a large ribonucleoprotein complex that orchestrates pre-mRNA splicing, and pharmacological targeting of the spliceosome has emerged as a potentially effective anticancer strategy. Several natural products inhibit RNA splicing by binding distinct spliceosomal subunits. Among them, thailanstatin A is a representative compound that suppresses splicing by engaging the SF3b subunit and thereby blocking the RNA splicing process [96] (Figure 41).

Thailanstatin A lacks an obvious functional handle suitable for linker installation. To address this limitation, researchers coupled its carboxylic acid to ethylenediamine, thereby introducing an amine-bearing spacer that is commonly used as an attachment point for linker conjugation [96]. A second major challenge in developing this natural product as an ADC payload arises from the presence of multiple reactive functionalities within its structure. For example, the diene motif in the central core can undergo a Diels–Alder reaction with the maleimide groups frequently used in bioconjugation, potentially leading to undesired side reactions. This issue was mitigated by replacing the maleimide handle with an alternative conjugation chemistry, such as a haloacetamide moiety. An ADC incorporating these two structural modifications together with a cleavable linker has been disclosed in patent literature as an early example of a thailanstatin-based payload, and it exhibited moderate activity across multiple HER2-positive cell lines.

**Figure 42:**
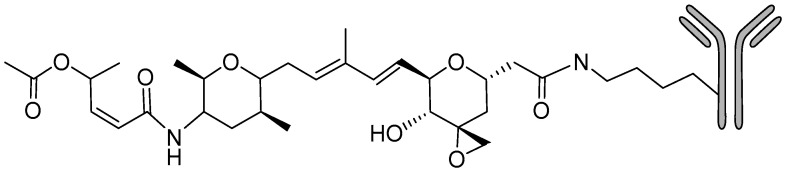
The structure of thailanstatin-based ADCs.

Recent work from Pfizer further showed that directly coupling the carboxyl group of thailanstatin A to lysine residues on the antibody surface, i.e., a linker-free lysine conjugation strategy, can yield the most potent thailanstatin-based ADCs reported to date (Figure 42). Unlike many other payload classes, the antitumor activity of these lysine conjugates appears to be strongly dependent on the drug loading level (DAR). In a gastric cancer xenograft model, this ADC demonstrated robust therapeutic efficacy.

#### 6.3.3. NAMPT Inhibitors

Nicotinamide phosphoribosyltransferase (NAMPT) is a key rate-limiting enzyme in the NAD^+^ salvage pathway that catalyzes the conversion of nicotinamide to nicotinamide mononucleotide (NMN). NAMPT inhibitors have demonstrated antitumor activity in multiple preclinical studies and clinical evaluations [97,98,99,100]. However, their clinical utility has been constrained by on-target toxicities and dose-limiting adverse events, including thrombocytopenia and gastrointestinal toxicities, among others.

**Figure 43:**
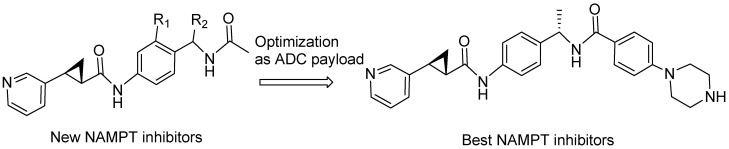
The structure of NAMPT inhibitors.

Researchers at Novartis identified a suitable conjugation handle on a NAMPT-inhibitor payload by introducing a piperazine moiety at the para position of the phenyl ring, thereby establishing a well-defined attachment site for linker installation. The resulting modified payload retained nanomolar potency in c-Kit- and HER2-positive cell lines and exhibited a favorable tolerability profile. In vivo, the corresponding ADC showed target-dependent antitumor activity, supporting the feasibility of NAMPT inhibitors as selectively delivered payloads (Figure 43).

#### 6.3.4. Proteasome Inhibitors: Kalamycins

**Figure 44:**
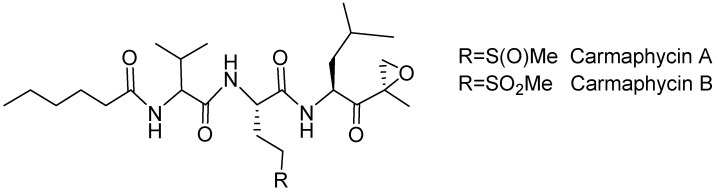
Structures of carmaphycin A and carmaphycin B, highlighting the methionine sulfoxide/sulfone region relevant to subsequent payload-optimization strategies.

Researchers have isolated two novel proteasome inhibitors, carmaphycin A and carmaphycin B, from a Curaçao cyanobacterium. Both compounds feature a leucine-derived α,β-epoxyketone warhead that is directly linked to either a methionine sulfoxide or methionine sulfone moiety (Figure 44). These molecules were shown to inhibit the β5 subunit of the Saccharomyces cerevisiae 20S proteasome (the chymotrypsin-like activity) and exhibited potent cytotoxicity against lung and colon cancer cell lines [101].

However, owing to their very high intrinsic potency, carmaphycin-class compounds often exhibit limited selectivity and may therefore be associated with substantial toxicity. Consequently, highly cytotoxic carmaphycin derivatives are well suited to serve as ADC warheads, where the required potency can be retained while targeted delivery improves tolerability by restricting exposure in normal tissues. In the design of carmaphycin-based payload analogues, a common strategy has been to introduce a methionine sulfone derivative at the P2 position (as in carmaphycin B), rather than the methionine sulfoxide present in carmaphycin A. This modification avoids the added structural complexity arising from mixtures of sulfoxide stereoisomers.

First-generation carmaphycin analogues introduced an amine handle at the P4 terminus, but this attachment site proved suboptimal and was associated with a marked reduction in payload cytotoxicity. In second-generation analogues, an amine was instead installed on the P2 side chain, with the most favorable results achieved by sulfonyl-linked extension of a short ethylamine substituent. Third-generation designs further connected the sulfone and amine through an aryl linker, thereby reducing overall molecular basicity. Both second- and third-generation payloads retained strong in vitro activity and were successfully coupled to either cleavable or non-cleavable linkers. Nevertheless, the resulting ADCs did not surpass trastuzumab in tumor cell-killing activity across the evaluated cancer cell lines.

#### 6.3.5. RNA Polymerase II Inhibitors: Amatoxins

In the context of ADC technology, the use of amatoxins, potent transcription inhibitors, as payloads is a relatively new direction. Nine naturally occurring amatoxins share a common core structure: a bicyclic octapeptide composed of eight L-amino acids forming a macrocycle, with a sulfoxide bridge between tryptophan and cysteine residues. Three hydroxyl-bearing side chains confer good water solubility and play crucial roles in binding to RNA polymerase II. Among these, α-amanitin and β-amanitin account for approximately 90% of natural amatoxins.

**Figure 45:**
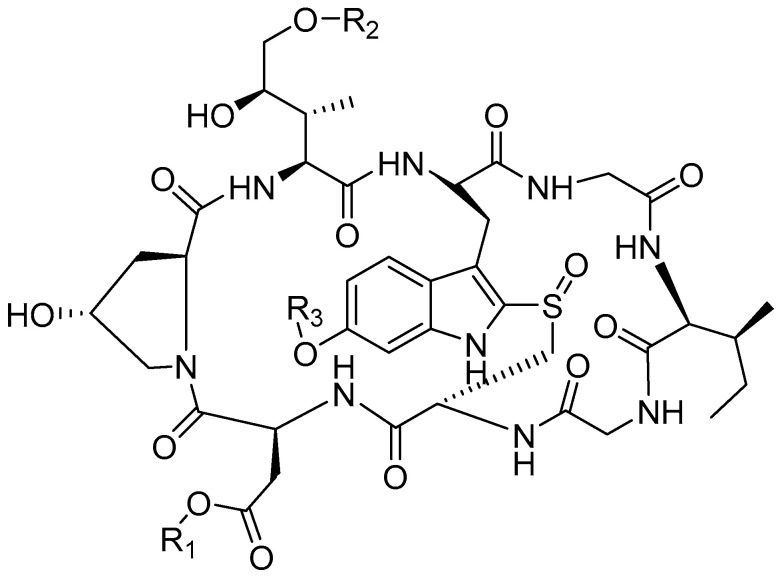
Representative conjugation sites on the amatoxin scaffold that can be exploited for linker-mediated coupling to antibodies.

To date, three main amatoxin conjugation strategies have been developed for ADC construction. The first involved coupling the carboxyl group of β-amanitin to lysine residues on IgG. Although this approach yielded ADCs with good plasma stability and high cytotoxicity, the overall bioconjugation efficiency was low. The second strategy utilized the hydroxyl group of dihydroisoleucine as the attachment site; a glutathione-based linker was introduced at this position and then coupled to lysine residues, producing ADCs with excellent in vitro cytotoxicity and in vivo antitumor activity. However, serum carboxylesterases could cleave the linker, compromising circulatory stability. The third and now standard approach uses the 6-hydroxyl group of tryptophan as the conjugation site. Etherification of this phenolic position with various linker motifs produces ADCs with high stability and potency. Other amino acids in the amatoxin scaffold, such as hydroxyproline, glycine, isoleucine and cysteine, are either chemically inert or essential for binding RNA polymerase II and are therefore unsuitable for modification (Figure 45).

**Figure 46:**
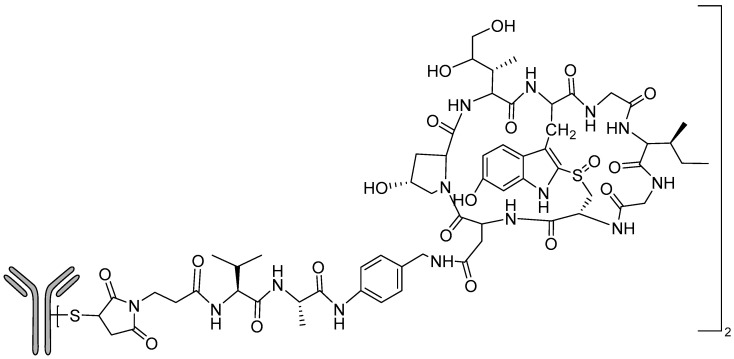
Structure of the amanitin-based ADC HDP-101 and its linker–payload design.

A representative amatoxin-based ADC is HDP-101, which uses a synthetic amanitin analog optimized for enhanced stability (Figure 46). Compared with natural amatoxins, this analog lacks the 6′-hydroxyl group on tryptophan and replaces the sulfoxide bridge with a thioether. A cathepsin B-cleavable linker is attached via an amide bond to the side chain of an aspartate residue.

Recently, Park and co-workers developed OHPAS, a novel diaryl sulfate linker motif for phenol-containing payloads. In this design, one aryl group is derived from the phenolic payload and the other from a phenolic linker unit. Application of OHPAS chemistry to trastuzumab–amanitin ADCs yielded constructs with strong cytotoxicity in vitro and in vivo, further expanding the linker repertoire for amatoxin-based ADCs.

### 6.4. Innovative Payload Modalities

Beyond conventional cytotoxic small molecules, multiple emerging payload modalities are being explored to further broaden mechanisms of action and improve therapeutic indices, including targeted protein degradation (PROTACs), peptide–drug conjugates (PDCs), near-infrared photoimmunotherapy (NIR-PIT), radionuclide drug conjugates (RDCs), and immune agonist payloads.

#### 6.4.1. PROTAC-Based Payloads

Antibody-PROTAC conjugates represent an emerging therapeutic modality that integrates the antibody–drug conjugate (ADC) paradigm with proteolysis-targeting chimera (PROTAC) technology. In this design, the antibody’s high target specificity is leveraged to selectively deliver a PROTAC payload, endowed with catalytic protein degradation capability, into defined cell populations. Unlike conventional ADCs that typically employ cytotoxic small-molecule payloads, these conjugates use PROTACs as the effector moiety to eliminate disease-relevant proteins via the ubiquitin–proteasome system. Mechanistically, the conjugate first recognizes and binds a specific antigen on the target cell surface; the resulting complex is then internalized via endocytosis, followed by lysosomal processing that cleaves the linker and releases the PROTAC intracellularly. The liberated PROTAC subsequently functions catalytically by recruiting an E3 ubiquitin ligase to form a ternary complex with the target protein, promoting target ubiquitination and directing it to proteasomal degradation. Owing to the catalytic, event-driven nature of PROTACs, substoichiometric amounts can induce sustained target depletion through multiple degradation cycles.

**Figure 47:**
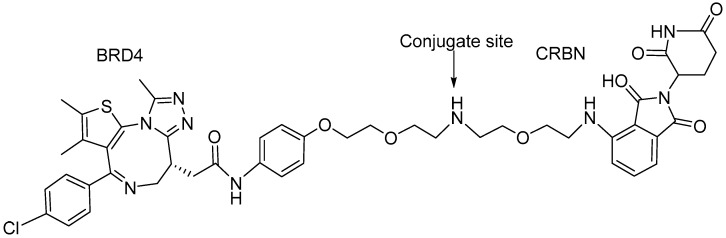
Representative PROTAC payload structures used in the design of antibody–PROTAC conjugates.

This strategy has the potential to address several limitations of current therapeutic approaches. First, antibody-mediated targeted delivery can increase PROTAC accumulation in diseased tissue while reducing exposure in normal organs, thereby improving the anticipated safety profile. Second, because its pharmacology is event-driven, aimed at eliminating the protein rather than continuously inhibiting its activity, this approach may expand the range of tractable targets, including those traditionally regarded as “undruggable.” For example, conjugation of a BRD4-directed PROTAC to an anti-HER2 antibody can yield a macromolecular construct of approximately 150 kDa, with an average drug-to-antibody ratio (DAR) of 4 PROTAC units per antibody (Figure 47). This design is intended to enable selective, intracellular degradation of BRD4 in HER2-positive tumor cells, combining tumor-targeting specificity with catalytic target depletion and thus demonstrating promising translational potential. Figure 47 shows representative PROTAC payload structures rather than the full chemical structure of the antibody–PROTAC conjugate.

#### 6.4.2. Peptide–Drug Conjugates (PDCs)

Peptide–drug conjugates (PDCs) are an emerging class of targeted therapeutics that typically comprise three key elements: a homing peptide, a cytotoxic payload, and a linker that covalently couples the two. Relative to antibody–drug conjugates, PDCs offer several potential advantages, including smaller molecular size, enhanced penetration into tumor tissue, reduced immunogenicity, and cost-effective large-scale manufacture via solid-phase peptide synthesis. In addition, PDCs may exhibit favorable pharmacokinetic tunability and improved batch-to-batch uniformity, collectively positioning them as promising next-generation targeted anticancer agents. Depending on the specific homing peptide and linker design, PDCs generally operate via two principal mechanisms. In one mode, the intact conjugate is internalized through receptor-mediated endocytosis, followed by intracellular payload release to elicit cytotoxicity. In the other, the conjugate undergoes selective cleavage within the tumor microenvironment, for example, by tumor-associated enzymes or microenvironmental conditions, thereby releasing the payload extracellularly, which is subsequently taken up by tumor cells to exert its therapeutic effect.

To date, only two PDC products have achieved regulatory approval worldwide. Among them, Lutathera (^177^Lu-DOTATATE), a radiolabeled somatostatin-analog peptide, has been approved for the treatment of gastroenteropancreatic neuroendocrine tumors (GEP-NETs) by targeting somatostatin receptors [102]. This product reportedly generated approximately USD 605 million in revenue in 2023. In contrast, Pepaxto (melphalan flufenamide) has been withdrawn from the market, largely due to insufficient targeting selectivity and an unfavorable benefit–risk profile relative to available alternatives.

#### 6.4.3. Near-Infrared Photoimmunotherapy (NIR-PIT) Payloads

Near-infrared photoimmunotherapy (NIR-PIT) is an emerging targeted anticancer platform whose core components include a tumor-specific monoclonal antibody, a photoactivatable agent, and a linker that covalently couples the two, thus positioning NIR-PIT conceptually within the antibody–drug conjugate (ADC) framework. This modality is implemented in conjunction with a near-infrared light irradiation device: the antibody enables selective delivery and accumulation at tumor sites, after which localized near-infrared illumination activates the conjugated photosensitizer to elicit a highly specific biophysical effect. In doing so, NIR-PIT can achieve potent tumor cell eradication while maximally preserving surrounding normal tissues.

The mechanism of NIR-PIT is characterized by a dual mode of action. First, upon near-infrared light activation, the conjugate induces rapid, localized destruction of antibody-bound tumor cells, leading to efficient target cell killing. Second, the resulting cell death can be accompanied by immunogenic cell death (ICD), which promotes the release of tumor antigens and danger-associated signals, thereby eliciting polyclonal antitumor immune responses. This immune amplification may facilitate the clearance of residual malignant cells that escape direct phototoxicity due to heterogeneous antigen expression, non-uniform drug distribution, or suboptimal light dose, ultimately enhancing overall therapeutic efficacy. In addition, NIR-PIT is amenable to combination with established immune checkpoint inhibitors (e.g., anti–PD-1, anti–PD-L1, or anti–CTLA-4 antibodies), which can further potentiate antitumor immunity and improve treatment outcomes.

**Figure 48:**
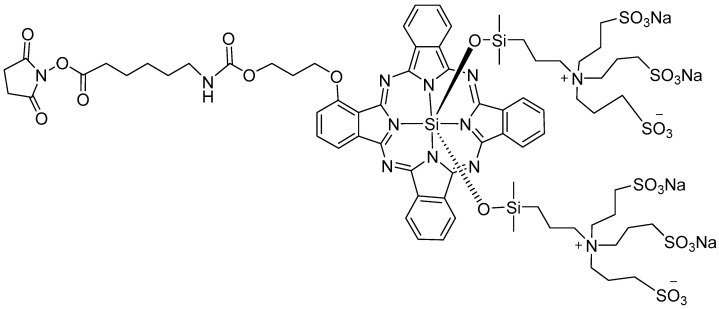
Chemical structure of IRDye700DX (128).

In contrast to conventional ADCs, the “payload” employed in NIR-PIT is not a classical cytotoxic small molecule but rather a photosensitizing agent, exemplified by the water-soluble phthalocyanine derivative IRDye700DX (IR700) (Figure 48). Following antibody binding to tumor-associated antigens, near-infrared irradiation triggers photochemical conversion of IR700, accompanied by cleavage/release of its hydrophilic side chains and a consequent abrupt increase in hydrophobicity. This physicochemical shift perturbs membrane integrity, culminating in rapid, highly selective immunogenic cell death (ICD) and tumor-restricted cytotoxicity. Although the clinical and commercial prospects of photoimmunotherapeutic ADC-like constructs remain to be fully established, the development of new targets and next-generation photoactivatable effectors based on this principle may open a promising avenue for innovation within the ADC landscape.

Near-infrared photoimmunotherapy is a targeted anticancer platform in which a tumor-specific antibody is conjugated to a photoactivatable agent and activated locally by near-infrared light. In contrast to conventional ADCs, the “payload” is typically a photosensitizer (e.g., IR700 derivatives) rather than a classical cytotoxin. After antibody binding, light exposure triggers rapid, spatially confined biophysical damage to cancer cells, while minimizing injury to surrounding normal tissues.

NIR-PIT can also induce immunogenic cell death, thereby promoting antitumor immune responses. This creates opportunities for combination with immune checkpoint inhibitors (e.g., anti-PD-1/PD-L1 or anti-CTLA-4) to amplify adaptive immunity and improve durable control.

#### 6.4.4. Radionuclide Drug Conjugates (RDCs)

Radiopharmaceutical drug conjugates (RDCs) can exert therapeutic activity without requiring cellular internalization, and they are often engineered with non-cleavable architectures to enhance stability in systemic circulation. In addition, the radionuclides carried by RDCs may be preferentially retained within the tumor microenvironment, which can reduce off-target irradiation of normal tissues and confer a distinctive safety advantage [103]. Structurally, RDCs are typically composed of three core elements: (i) a targeting moiety, most commonly a monoclonal antibody or a small-molecule ligand that selectively recognizes tumor-associated markers; (ii) a linking/chelating module, comprising a covalent linker that couples the targeting unit to a chelator, together with the chelator that coordinates and stabilizes the radionuclide through metal–ligand interactions; and (iii) an effector component, namely a radionuclide selected for therapeutic or diagnostic purposes based on its decay characteristics and emission profile [104]. This modular design enables precise tumor targeting and controllable radiologic effects.

Depending on the selected effector module, RDCs can be broadly categorized into diagnostic and therapeutic constructs. Diagnostic RDCs are primarily developed for molecular imaging, most commonly PET and SPECT, and therefore employ radionuclides that emit positrons (β^+^ emitters) for PET or single photons (γ emitters) for SPECT. Representative diagnostic radionuclides include ^68^Ga, ^18^F, ^64^Cu, ^89^Zr, ^99m^Tc, and ^111^In. In contrast, therapeutic RDCs are designed to deliver cytotoxic radiation capable of inducing DNA double-strand breaks, eradicating hypoxic tumor cell populations, and providing a cross-fire effect that can extend radiation dose beyond antigen-positive cells to partially address intratumoral heterogeneity. Accordingly, therapeutic radionuclides are typically long-range β^−^ emitters and/or high–linear energy transfer (LET) α emitters, with representative examples including ^225^Ac, ^223^Ra, ^213^Bi, ^211^At, ^177^Lu, ^90^Y, ^131^I, and ^188^Re [30,105].

The development of radiopharmaceuticals is increasingly shifting from agents with a single diagnostic or therapeutic function toward an integrated theranostic paradigm. Radionuclides such as ^64^Cu and ^177^Lu have attracted considerable attention because they can support both molecular imaging and radionuclide therapy, and their clinical exploration has expanded beyond conventional oncology applications to emerging indications, including neurodegenerative disorders. In terms of China’s domestic innovation, ^131^I-metuximab (Licartin) represents a landmark achievement as the world’s first approved radiolabeled antibody conjugate for the treatment of hepatocellular carcinoma [106]. In addition, next-generation candidates such as ^177^Lu-EB-FAPI have advanced into Phase III clinical evaluation, underscoring the sustained innovation and translational momentum in this field [107,108].

#### 6.4.5. Immune Agonists (TLR and STING) as Payloads

Immunostimulant antibody conjugates (ISACs) represent an emerging immunotherapeutic modality designed to achieve precise immune activation by coupling an immunomodulatory agonist payload to a tumor-targeting antibody. Distinct from conventional ADCs that primarily rely on direct cytotoxicity, ISACs are proposed to operate through a multistep mechanism: (i) the antibody moiety selectively recognizes and binds tumor-associated antigens; (ii) the resulting immune complex is internalized by antigen-presenting cells (APCs) via Fc receptor-mediated uptake; and (iii) within APCs, the conjugated agonist engages and activates Toll-like receptor (TLR) signaling pathways [109,110]. This design enables preferential delivery of immune agonists to the tumor microenvironment, thereby initiating innate immune activation and subsequently promoting adaptive antitumor immune responses. By restricting immunostimulatory activity to the intended site of action, ISACs may mitigate the systemic toxicities commonly associated with the systemic administration of immune stimulants, improving therapeutic efficacy while reducing the risk of widespread adverse events [109,110].

As an emerging therapeutic strategy, immunostimulant antibody conjugates (ISACs) have predominantly employed IgG1 isotype antibodies as the delivery scaffold. IgG1 offers favorable developability attributes, including high structural stability and a prolonged serum half-life, which help sustain therapeutically relevant systemic exposure. Moreover, IgG1 exhibits strong affinity for Fc gamma receptors (FcγRs), which are broadly expressed on multiple immune cell subsets; FcγR engagement can facilitate the recruitment and activation of immune cells within the tumor locale, thereby supporting immune-mediated antitumor activity [110].

With respect to payload design, ISACs most commonly incorporate small-molecule immunomodulators, such as Toll-like receptor (TLR) agonists (e.g., TLR7/8/9 agonists) or stimulator of interferon genes (STING) agonists. These immune-activating payloads are intended to reprogram the tumor immune microenvironment and amplify host antitumor immunity, addressing a key limitation of traditional ADCs that primarily rely on direct cytotoxic mechanisms rather than immune potentiation. Despite these mechanistic advantages, ISACs remain at an early stage of development overall. Compared with ADCs, ISACs pose more intricate challenges in optimizing the efficacy–safety balance, given the need to tightly control the magnitude, location, and duration of immune activation [110,111].

## 7. Challenges and Future Directions

Despite significant progress, ADC development faces several critical challenges. Off-target toxicity, caused by premature payload release or cross-reactivity with normal tissues expressing low levels of TAAs, leads to toxicities such as myelosuppression, neuropathy, and cardiotoxicity. Tumor heterogeneity and acquired resistance, resulting from variable antigen expression, impaired internalization, or payload efflux via P-glycoprotein, limit efficacy. Pharmacokinetic limitations, including heterogeneous DAR, rapid clearance, and immune-mediated ADC degradation, affect therapeutic consistency. Manufacturing complexity, particularly for site-specific conjugation and quality control, leads to high production costs.

To address these challenges, several innovative strategies are being explored. Bispecific/multispecific ADCs target two or more antigens to enhance tumor specificity and overcome heterogeneity. Smart linkers, activated by tumor-specific signals (e.g., hypoxia, reactive oxygen species, extracellular proteases), improve payload release specificity. Novel payload classes, including immunomodulators (e.g., TLR agonists, PD-1 inhibitors), trigger antitumor immunity and expand therapeutic scope. Combination therapies, combining ADCs with immune checkpoint inhibitors, chemotherapy, or radiotherapy, enhance synergistic antitumor effects [111]. Personalized medicine, identifying predictive biomarkers (e.g., antigen expression, linker-cleaving enzyme levels), selects patients most likely to benefit from ADC therapy.

## 8. Conclusions

Antibody–drug conjugates represent a rapidly evolving class of targeted anticancer agents that have transformed the treatment of hematological malignancies and solid tumors. Through rational design of antibodies, linkers, and payloads, ADCs achieve precise tumor targeting and potent cytotoxicity, offering new hope for patients with refractory cancers. The evolution from first-generation to third-generation ADCs has addressed key limitations, with site-specific conjugation, optimized linkers, and ultra-potent payloads driving clinical success. However, challenges such as off-target toxicity, drug resistance, and manufacturing complexity remain. Future advancements in bispecific ADCs, smart linkers, novel payloads, and combination therapies are expected to further improve efficacy and safety. As our understanding of ADC biology and tumor heterogeneity deepens, ADCs will continue to play a central role in precision oncology, paving the way for more effective and personalized cancer treatments.

## Figures and Tables

**Table 1:** Approved Antibody–Drug Conjugates (ADCs) and Their Key Characteristics.

No.	Antibody–Drug Conjugate (Developer)	Trade Name	Target Antigen	Linker	Cytotoxic Payload	Payload Action	Drug Antibody Ratio (DAR)
1	Ado-trastuzumab emtansine	Kadcyla	HER2	SMCC	DM1	Microtubule Inhibitor	3.5
2	Mirvetuximab soravtansine (ImmunoGen)	Elahere	FRα	sulfo-SPDB	DM4	Microtubule Inhibitor	3.4
3	Brentuximab vedotin (Seagen)	Adcetris	CD30	mc-VC-PABC	MMAE	Microtubule Inhibitor	4
4	Polatuzumab vedotin	Polivy	CD79B	mc-VC-PABC	MMAE	Microtubule Inhibitor	3.5
5	Enfortumab vedotin (Seagen)	Padcev	Nectin-4	mc-VC-PABC	MMAE	Microtubule Inhibitor	3.8
6	Disitamab vedotin	Aidixi	HER2	mc-VC-PABC	MMAE	Microtubule Inhibitor	4
7	Tisotumab vedotin (Genmab/Seagen)	Tivdak	TF	mc-VC-PABC	MMAE	Microtubule Inhibitor	4
8	Belantamab mafodotin (GSK)	Blenrep	BCMA	mc	MMAF	Microtubule Inhibitor	4
9	Loncastuximab tesirine (ADC Therapeutics)	Zynlonta	CD19	dipeptide	PBD dimer	DNA Cleavage	2.3
10	Gemtuzumab ozogamicin	Mylotarg	CD33	hydrazone	N-acetyl-γ-calicheamicin	DNA Cleavage	2~3
11	Inotuzumab ozogamicin	Besponsa	CD22	hydrazone	N-acetyl-γ-calicheamicin	DNA Cleavage	5~7
12	Fam-trastuzumab deruxtecan	Enhertu	HER2	tetrapeptide	DXd	TOP1 Inhibitor	7~8
13	Sacituzumab tirumotecan	JIa	Trop-2	methyl sulfonyl pyrimidine	KL610023	TOP1 Inhibitor	7.4
14	Sacituzumab govitecan (Immunomedics)	Trodelvy	Trop-2	CL2A	SN38	TOP1 Inhibitor	7.6
15	Cetuximab sarotalocan	Akalux	EGFR	NA	IRDye700DX	Membrance damage	1.3~3.8
16	Moxetumomab pasudotox	Lumoxiti	CD22	mc-VC-PABC	PE38	Protein synthesis inhibition	NA

## Data Availability

Not applicable.
